# A comparison of stomatal conductance responses to blue and red light between C3 and C4 photosynthetic species in three phylogenetically-controlled experiments

**DOI:** 10.3389/fpls.2023.1253976

**Published:** 2023-09-27

**Authors:** Emmanuel L. Bernardo, Cristina Rodrigues Gabriel Sales, Lucía Arce Cubas, Richard L. Vath, Johannes Kromdijk

**Affiliations:** ^1^Department of Plant Sciences, University of Cambridge, Cambridge, United Kingdom; ^2^Institute of Crop Science, College of Agriculture and Food Science, University of the Philippines Los Baños, College, Los Baños, Laguna, Philippines; ^3^Carl R. Woese Institute for Genomic Biology, University of Illinois at Urbana-Champaign, Urbana, IL, United States

**Keywords:** photosynthesis, blue light, red light, stomata, stomatal conductance, Cleomaceae, *Flaveria*, *Alloteropsis*

## Abstract

**Introduction:**

C_4_ photosynthesis is an adaptation that has independently evolved at least 66 times in angiosperms. C_4_ plants, unlike their C_3_ ancestral, have a carbon concentrating mechanism which suppresses photorespiration, often resulting in faster photosynthetic rates, higher yields, and enhanced water use efficiency. Moreover, the presence of C_4_ photosynthesis greatly alters the relation between CO_2_ assimilation and stomatal conductance. Previous papers have suggested that the adjustment involves a decrease in stomatal density. Here, we tested if C_4_ species also have differing stomatal responses to environmental cues, to accommodate the modified CO_2_ assimilation patterns compared to C_3_ species.

**Methods:**

To test this hypothesis, stomatal responses to blue and red-light were analysed in three phylogenetically linked pairs of C3 and C4 species from the Cleomaceae (Gynandropsis and Tarenaya), Flaveria, and Alloteropsis, that use either C3 or C4 photosynthesis.

**Results:**

The results showed strongly decreased stomatal sensitivity to blue light in C_4_ dicots, compared to their C_3_ counterparts, which exhibited significant blue light responses. In contrast, in C_3_ and C_4_ subspecies of the monocot *A. semialata*, the blue light response was observed regardless of photosynthetic type. Further, the quantitative red-light response varied across species, but the presence or absence of a significant stomatal red-light response was not directly associated with differences in photosynthetic pathway. Interestingly, stomatal density and morphology patterns observed across the three comparisons were also not consistent with patterns commonly asserted for C_3_ and C_4_ species.

**Discussion:**

The strongly diminished blue-light sensitivity of stomatal responses in C_4_ species across two of the comparisons suggests a common C_4_ feature that may have functional implications. Altogether, the strong prevalence of species-specific effects clearly emphasizes the importance of phylogenetic controls in comparisons between C_3_ and C_4_ photosynthetic pathways.

## Introduction

Efficient coordination of carbon gain versus water loss by stomata is achieved by a range of responses to environmental factors, most notably light and CO_2_. Light stimulates stomatal opening via at least two distinct signaling pathways with contrasting action spectra (Shimazaki et al., 2007), which are either perceived directly in the guard cells (GCs) or indirectly via signals derived from the underlying mesophyll (Mott, 2009; Lawson et al., 2014).

Blue light (BL) is a potent signal for stomatal opening, and GCs contain all the components required for the blue light signaling pathway (Shimazaki et al., 2007; Suetsugu et al., 2014). Blue light activates rapid stomatal opening via a pathway that is independent of photosynthesis and saturates at relatively low fluence rates. Blue light triggers the autophosphorylation of phototropins, PHOT1 and PHOT2 via their substrate BLUS1 (BLUE LIGHT SIGNALING 1) (Inoue et al., 2008; Takemiya et al., 2013). This sequentially triggers the activation of plasma membrane H^+^ ATPase pump, which hyperpolarizes the plasma membrane, leading to the uptake of K^+^ through inward-rectifying channels. The accumulation of K^+^ and other solutes drives the influx of water into the GCs and the resulting turgor change leads to stomatal opening (Shimazaki et al., 2007).

Illumination with red light (or photosynthetically active wavelengths other than blue) also triggers stomatal opening but the quantum efficiency to stimulate opening is typically lower than blue light. An indirect effect of red light that is tied to intercellular CO_2_ (C_i_) is triggered by high (red) light through mesophyll photosynthesis, which reduces C_i_, and consequently affects g_s_. However, this feedback loop does not appear to be the only mesophyll-derived signal perceived by stomata. A significant response to red light was still observed in experiments where C_i_ was kept constant (Messinger et al., 2006). In addition, epidermal peel experiments revealed that GC responses to red light are reversibly altered by the presence of the underlying mesophyll tissue. Thus, an additional link between mesophyll photosynthesis and guard cell responses may exist (Lawson et al., 2014). The identity of the mesophyll signal however remains unresolved.

Plants with C_4_ photosynthesis are distinct from C_3_ plants in anatomy and biochemistry. C_4_ operates a CO_2_-concentrating mechanism (CCM) between two morphologically different cell types, bundle sheath cells (BSC) and mesophyll (M) cells (Sage, 2004). Having a CCM alters the relationship of photosynthesis with CO_2_ in C_4_ species which confers them a photosynthetic advantage over C_3_ species under saturating light conditions and hot environments (Bowyer and Leegood, 1997). In these environments, C_4_ species typically display higher intrinsic water use efficiency and net assimilation rates (Sage, 2004) compared to C_3_ species (Lambers and Oliveira, 2019). It can therefore be argued that differential stomatal regulation is required to optimize photosynthetic capacity and water use efficiency between C_3_ and C_4_ species. And that the mesophyll signal argued above may be different between C_3_ and C_4_ species. However, not much is known about stomatal sensitivities with regard to photosynthetic types under various light and CO_2_ environments.

Comparisons between C_4_ and C_3_ species can easily be confounded by phylogenetic distance between the species, which would lead to differences not necessarily associated with the photosynthetic pathway (Monson, 1996; Taylor et al., 2010). For instance, contrasting blue light responses in C_3_ and C_4_ were observed in crop species (Zhen and Bugbee, 2020), but the findings could not be directly linked to photosynthetic type alone because the species examined were separated by considerable phylogenetic distance. Closely related species or even subspecies with differing photosynthetic pathways offer a way around this issue, allowing a comparison between photosynthetic types while controlling for evolutionary distance (Monson, 1996; Huxman and Monson, 2003).

In this study, blue and red-light stomatal responses were studied in three phylogenetically-controlled experiments, using closely related species from the Cleomaceae (*Gynandropsis* and *Tarenaya*), *Flaveria*, and *Alloteropsis*, that use either C_3_ or C_4_ photosynthesis. The results show a striking lack of sensitivity to blue light in the C_4s_ *Gynandropsis gynandra*, and *F. trinervia* and *F. bidentis*, compared to their C_3_ counterparts *Tarenaya hassleriana*, and *F. cronquistii* and *F. robusta*, which exhibited significant blue light responses. In contrast, in *A. semialata*, the blue light response was observed regardless of photosynthetic type. The quantitative red-light response varied between species, however the presence or absence of a significant stomatal red-light response was not related to differences in photosynthetic pathway.

## Materials and methods

### Plant materials

Stomatal responses were compared across closely related C_3_ and C_4_ species from the Cleomaceae (Cleomaceae), Asteraceae (*Flaveria*), and Poaceae (*Alloteropsis*), with considerable phylogenetic distance separating each of the controlled comparisons. From the genus Cleomaceae, *Tarenaya hassleriana* (C_3_) was compared with *Gynandropsis gynandra* (C_4_, NAD-ME subtype). Seeds of C_3_ *T. hassleriana* (Cleomaceae) were obtained from Prof. Julian Hibberd’s group (Cambridge, UK). From the genus *Flaveria*, *F. trinervia* and *F. bidentis* (C4-NADP-ME) were initially compared with *Flaveria pringlei* as the C_3_ representative. However, the *F pringlei* accession that most manuscripts have described and was used here, likely has seen some hybridization with *Flaveria angustifolia* in its history, which is a C3-C4 intermediate. We therefore decided to do a second set of experiments using *F. cronquistii* and *F.robusta* as ‘true’ C3 species in addition to the three aforementioned species. Seeds of *F. bidentis*, *F. trinervia*, and *F. pringlei* were a kind gift from Dr. Peter Westhoff (University of Dusseldorf, Germany). Clonal plants of *F. cronquistii* and *F.robusta* were a gift from Dr. Marjorie Lundgren (Lancaster University, UK) and Prof. Julian Hibberd (Cambridge University, UK), respectively. Finally, from the genus *Alloteropsis*, *A. semialata* subsp. eckloniana GMT (C_3_) and *A. semialata* subsp. semialata MDG and MAJ (both C_4,_ mixed NADP-ME-PEPCK subtypes) were a gift from Prof. Pascal-Antoine Christin (University of Sheffield, UK).

### Growth conditions

Individual seedlings of *G. gynandra* and *T. hassleriana* were grown from seeds and transferred in 10 cm H x 9 cm L x 9 cm W plastic pots with M3 compost (Levington^®^, Scotts, Ipswich, UK). Seeds of C_4_ *F. bidentis*, C_4_ *F. trinervia*, and C_3_-like *F. pringlei* were also germinated on M3 compost. The seedlings and clonal plants of C_3_ *F. cronquistii* and C_3_ *F.robusta* propagated from cuttings were grown in 10 cm H x 9 cm L x 9 cm W plastic pots containing M3 compost.

The Cleomaceae and *Flaveria* species were grown in an environmentally-controlled growth room at the Plant Growth Facility (PGF), Department of Plant Sciences, University of Cambridge. Photoperiod was kept at 16 hours (6:00 to 22:00) of light supplied by fluorescent lamps with a photosynthetic photon flux density (PPFD) of 300 µmol m^-2^ s^-1^. The growth room was maintained at day and night temperatures of 20°C and 60% RH at ambient CO_2_.

The *Alloteropsis* accessions were grown at the Cambridge Botanic Gardens Glasshouse. Plants were vegetatively propagated to contain 2-3 tillers per 1 L pots filled with 4:1 M3 compost: coarse vermiculite and 5 g controlled-release fertilizer (Osmocote^®^, Scotts Miracle-Gro Company, Marysville, OH, USA). Supplemental light from sodium halide lamps (140-160 W m^-2^) was provided from 4:00 to 20:00. Greenhouse set point temperature was at 18°C, while nighttime temperatures were at 15°C. CO_2_ and RH were at ambient.

### Gas exchange measurements

An open gas exchange system (LI-6400XT, LI-COR, Lincoln, NE, USA) with an integrated fluorometer and light source (LI-6400-40, LI-COR) was used to measure stomatal conductance (g_s_) and CO_2_ assimilation rate (*A*) at a range of different conditions as specified below.

All gas exchange data were collected from the youngest expanded leaf from plants with 4-5 sets of mature leaves.

### Experimental design

#### Red and blue light switchover experiment

To characterize stomatal responses to blue light, a series of switchover protocols were designed comprising sequential changes between 100% red (R) and 75% red + 25% blue light (RB), while keeping the total PPFD equal. Peak wavelengths for R and B lights in the LI-6400XT instruments are 635 and 465 nm, respectively. In Sequence R→RB→R, leaves were first acclimated to steady-state under 100% red light, and then switched to 75% red and 25% blue light, and then back to the original light environment of 100% red light. In some cases the complementary switchover experiment RB→R→RB was performed, and in this case, leaves were first acclimated to steady-state under 75% red and 25% blue light. 500 µmol m^-2^ s^-1^ PPFD was maintained throughout the time course for this experiment. The choice for 75% red + 25% blue was based on the fact that 125 µmol m^-2^ s^-1^ is sufficient to saturate the stomatal blue light response, and red background light is often found to enhance the blue light response. Each switchover experiment was initiated when steady state was reached (typically after 2 h). Gas exchange measurements were logged every 60 s throughout the protocol. After logging the first 5 min (in Cleomaceae and *Alloteropsis*) and 10 min (in *Flaveria*) of the steady state condition, the light composition was switched to either red or red+blue, followed finally by a switch back to the initial light composition. During each phase gas exchange parameters were logged for 60 mins (in the Cleomaceae and *Alloteropsis*) and 50 mins (in *Flaveria*). Thus, for each measurement series, 125 mins were recorded for Cleomaceae and *Alloteropsis* subspecies and 100 mins for *Flaveria*. The initial experiment in *Flaveria* was performed using C_3_-like *F. pringlei*, C_4_ *F. trinervia* and C_4_ *F. bidentis*. However, because C_3_-like *F. pringlei* has proto-Kranz (Sage, 2016), it is not considered a canonical C_3_. Therefore, a repeat experiment in *Flaveria* was performed to include two true C_3_ species, *F. cronquistii* and *F. robusta*. For this experiment, only sequence R*→*RB*→*R was repeated, since the previous experiments had shown that both sequences yielded very similar results. Reference CO_2_ during the experiment was controlled at 400 µmol mol^-1^, block temperature was kept at 25°C and average VPD was ca. 1.2 kPa. All measurements were taken between 10:00 and 16:00 h.

### Determination of the red-light response under constant C_i_

To characterize the red-light response of stomatal conductance, the protocol by Messinger et al. (2006) was adopted. This protocol measures the stomatal response to red light, while controlling for the potentially confounding effect of the stomatal CO_2_ response by maintaining a constant intercellular CO_2_ concentration (C_i_) at each light intensity. Briefly, the Messinger protocol involved illuminating the leaf segment clamped inside the cuvette with 800 µmol m^-2^ s^-1^ red-light to steady state. Subsequently, the PPFD was lowered step-wise from 800, 600, 400, and 200 µmol m^-2^ s^-1^, each change was initiated once steady state of stomatal conductance was achieved. As *A* and g_s_ changed in response to the new light environment, CO_2_ was manually adjusted using the mixer millivolt signal to keep C_i_ at the value observed at 800 µmol m^-2^ s^-1^.

### Stomatal morphology and density using epidermal impressions

Epidermal imprints from all species, except for *Flaveria trinervia*, were taken from adaxial and abaxial layers using clear nail varnish (Rimmel, London) using the same leaves used for gas exchange measurements. Dried nail varnish was lifted from the leaf using sticky tape (Sellotape) and affixed onto glass slides. Images were captured using a Nikon microscope (Olympus BX41, Japan) fitted with a CCD camera (U-TV0.5zc- 3/Micropublisher 3.3 RTV, Olympus, Japan) and installed with Q-Capture Pro 7 imaging software. Total magnifications of x200 and x400 (Olympus WHB 10x/20 eyepiece x Olympus PN 20x/0.40 or PCN 60x/0.80 objectives) were used for stomatal density counting and investigation of stomatal anatomical parameters, respectively, unless stated otherwise. For *Flaveria trinervia* the quality of nail varnish impressions was not sufficient to collect stomatal anatomical parameters, data were instead collected from epidermal peels, using the same microscopy set up with a total magnification of x400. Therefore, these data were not from the same leaf as was used for gas exchange but instead were taken from other leaves at the same developmental stage.

To assess stomatal size (SS), guard cell width (GCW) and guard cell length (GCL) were measured using FiJi software (Schindelin et al., 2012). SS was calculated using the formula for the area of an ellipse. Stomatal density (SD) was calculated as the number of stomata per unit area (mm^-2^). A stoma was counted if >50% was located within the field of view. Four random microscopic fields of view each from the abaxial and adaxial surfaces were collected from 3-5 biological replicates, giving >350 stomata per species, except C_3_ *F. cronquistii* and C_4_ *F. trinervia*.

### Statistical analysis

For the red and red+blue light switchover experiments, pairs of C_3_ and C_4_ species were measured in parallel between 10:00 and 16:00 h to control for the effect of circadian rhythms on stomatal conductance. Each phylogenetically-controlled comparison was an independent experiment. To estimate the impact of light on stomatal conductance resulting from the shift from one light environment to the next, a linear mixed-effects model was used. Light was the within-subject factor and species was the between-subject factor. Biological replicates were treated as random variables. To carry out the analysis, g_s_ and *A* at the introduction or removal of blue light (t = 5 min and t =65 min, R*→*BR*→*R) or the reverse (t = 5 and t = 65, BR*→*R*→*BR), and the final g_s_ at t = 125 min were extracted from the gas exchange time series. Planned contrasts were used to compare independent group means. Linear mixed-effects model was also fitted to the red-light response data set. Red-light PPFD and species were assigned as within- and between-subject variables, respectively, while individual biological replicates were treated as random variables. For every genus, the density of stomata on the abaxial and adaxial surfaces was analyzed using a two-way ANOVA, and planned contrasts were used to compare group means. Data on stomatal size were also analysed using ANOVA and *post-hoc* comparisons were carried out using Tukey’s HSD at an *α*=0.05. The statistical tests were carried out using R, version 4.2.1 (R Core Team, 2023) and RStudio, version 2023.6.0.421 (Posit Team, 2023) and JMP Pro 17 (SAS Institute). Graphs were plotted using R, version 4.2.1 and RStudio, version 2022.07.1 and the *ggplot2* package (Wickham, 2016). Figures were compiled using the *ggpubr* package (Kassambara, 2023).

## Results

### Stomatal sensitivity to blue light is lower in C_4_ species from the genus *Cleomaceae* and *Flaveria* compared to their C_3_ counterparts

**Figure 1:**
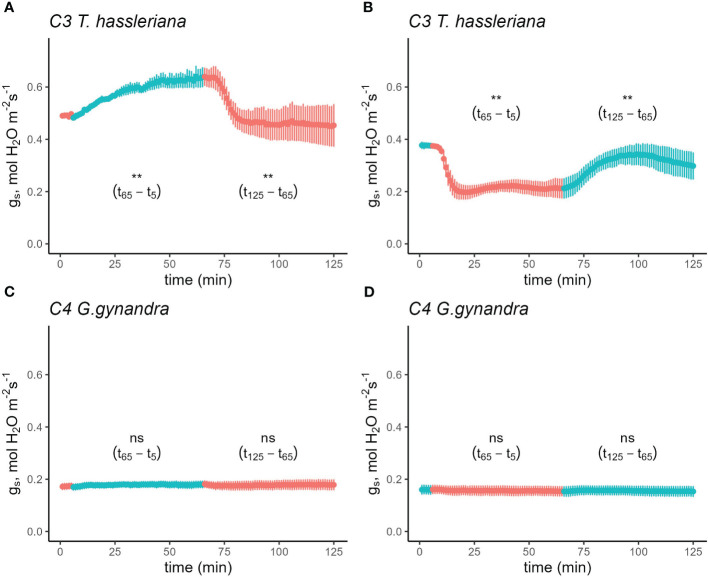
Time course of stomatal conductance (g_s_) response in C_3_ *T. hassleriana* **(A, B)** and C_4_ *G. gynandra* **(C, D)** in response to a sequence of 75% red + 25% blue light and vice versa. Leaves were initially acclimated in either 100% red **(A, C)** or 75% red+25% blue light **(B, D)** until steady-state was achieved (time = 0 in the figure). Subsequently, the light environment was switched depending on the initial light condition while maintaining a photosynthetic photon flux density of 500 µmol m^-2^ s^-1^. Subsequently, the leaves were acclimated to the new light condition for 1 h before returning it back to the original condition. Light conditions were reversed at t_6_ and t_65_. Reference CO_2_ was maintained at 400 µmol mol^-1^, block temperature was kept at 25°C and average VPD was 1.2 kPa. Data points represent mean ± se (n=4-5).

To compare the sensitivity of stomatal opening to blue light relative to red light of equal intensity between C_3_ and C_4_ species, gas exchange was measured in response to a sequence of 75% red:25% blue (RB) light to 100% red (R) with a total PAR of 500 µmol photons m^-2^ s^-1^ as well as the reverse sequence. Following steady state g_s_ of 0.49 mol H_2_O m^-2^ s^-1^ at R light, g_s_ in the C_3_ species *T. hassleriana* increased gradually to 0.64 mol H_2_O m^-2^ s^-1^ upon shifting to RB light and the effect was rapidly reversed when the light was returned to R (**Figure 1A** and **Table S1**). Similarly, when moving from RB→R in the reverse sequence, C_3_ *T. hassleriana* rapidly declined from 0.38 to 0.20 mol H_2_O m^-2^ s^-1^, and gradually increased again by the switch from R→RB over the course of an hour (**Figure 1B** and **Table S2**). In contrast, no effects of the R→RB→R sequence, nor the reverse RB→R→RB sequence were observed on g_s_ in the C_4_ species *G. gynandra*, which remained invariable at 0.19 mol H_2_O m^-2^ s^-1^ (**Figures 1C, D**; **Tables S1** and **S2**). It is also worth noting that the strong stomatal movements in C_3_ *T. hassleriana* did not correspond to parallel responses in *A* (**Figures S1A, B**; **Tables S3** and **S4**), which instead was not significantly impacted by the changes in light composition in either of the Cleomaceae species (**Figures S1C, D**; **Tables S1-S4**).

**Figure 2:**
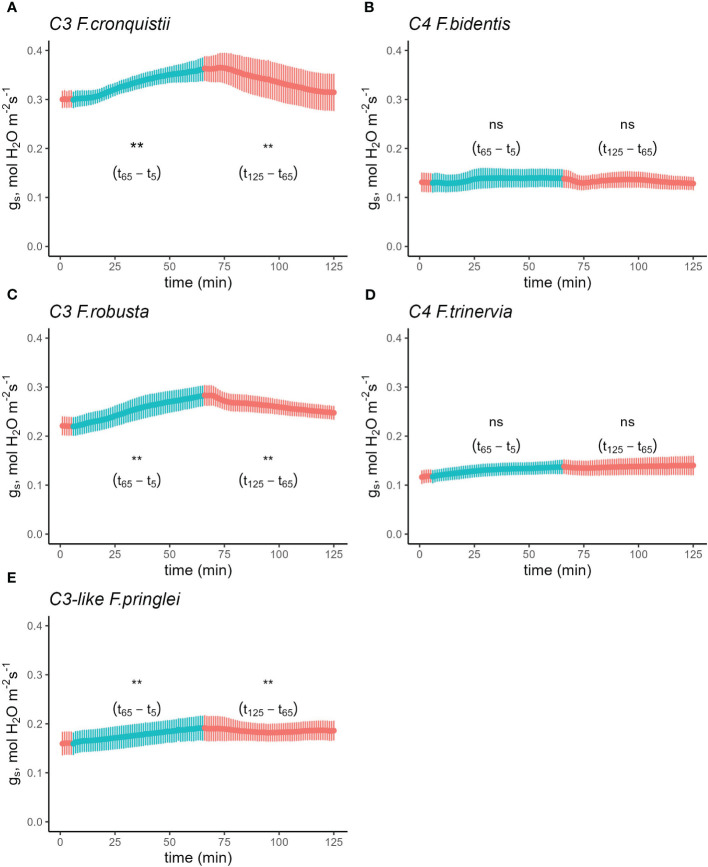
Time course of stomatal conductance (g_s_) response in C_3_ *F.robusta* **(A)**, C_3_ *F. cronquistii* **(B)**, C_4_ *F. trinervia* **(C)**, C_4_ *F. bidentis* **(D)**, and **(E)** C_3_-like *F. pringlei* in response to a sequence of 100% red and 75% red + 25% blue light. Leaves were initially acclimated under 100% red light until steady state was achieved (time = 0 in the figure). Subsequently, the light environment was switched to 75% red + 25% blue light while maintaining a photosynthetic photon flux density of 500 µmol m^-2^ s^-1^. The leaves were acclimated to the new light condition for 1 h before returning them back to the original condition for another hour before terminating the experiment. Light conditions were reversed at t_6_ and t_65_. Reference CO_2_ was maintained at 410 µmol mol^-1^, block temperature was kept at 25°C and average VPD was 1.2 kPa. Data points represent mean ± se (n=4-5). ** indicates significant difference at alpha = 0.05; ns, not significant.

To find out if these differences between C_3_ and C_4_ Cleomaceae species were representative of general differences in stomatal responses between photosynthetic pathways, the R*→*RB*→*R sequence was also performed on *Flaveria*, another genus which contains C_3_, C_3_-C_4_, and C_4_ species. The differences in blue light response between C_3_ and C_4_ species were also present in *Flaveria* (**Figure S2** and **Tables S5** and **S6**). However, to account for the fact that the first set of *Flaveria* experiments used the proto-Kranz C_3_-like species *F. pringlei* as the C_3_ representative, the experiment was repeated to include two other C_3_ *Flaveria* species, *F. robusta* and *F. cronquistii*. Since the complementary sequence RB→R→RB yielded the same results as the R→RB→R sequence, only the latter R→RB→R sequence was repeated (**Figure 2**) on two C_3_ species, *F. robusta* (**Figure 2A**) and *F. cronquistii* (**Figure 2B**), as well as the two C_4_ species, *F. trinervia* (**Figure 2C**) and *F. bidentis* (**Figure 2D**) and C_3_-like *F. pringlei* (**Figure 2E**). After reaching steady state in R, g_s_ increased significantly from 0.22 mol H_2_O m^-2^ s^-1^ to 0.28 (**Figure 2A**) mol H_2_O m^-2^ s^-1^ in *F. robusta* upon the switch to RB and from 0.30 mol H_2_O m^-2^ s^-1^ to 0.36 mol H_2_O m^-2^ s^-1^ in C_3_ *F. cronquistii* (**Figure 2B**). On the other hand, g_s_ in C_4_ *F. trinervia* showed only a slight increase from 0.11 mol H_2_O m^-2^ s^-1^ to 0.13 mol H_2_O m^-2^ s^-1^ under RB light (**Figure 2C**), and similarly g_s_ in C_4_ *F. bidentis* remained unchanged at 0.13 mol H_2_O m^-2^ s^-1^ (**Figure 2D**). Finally, in C_3_-like *F. pringlei*, from an initial g_s_ of 0.16 mol H_2_O m^-2^ s^-1^ it rose to 0.19 mol H_2_O m^-2^ s^-1^ under BR (**Figure 2E**). Further, the stimulating effects on g_s_ of the R→RB switch in the C_3_ and C_3_-like species were largely reversed by the switch back to R light, but again g_s_ remained unaffected in the C_4_ species (**Table S7**). The light composition switches marginally, but significantly affected the rate of net CO_2_ assimilation among the five species with slight lower rates of *A* under the RB part of the experiment (**Figure S3** and **Table S8**, p=0.0122).

### C_3_ and C_4_ subspecies of *A. semialata* display the canonical blue light response

**Figure 3:**
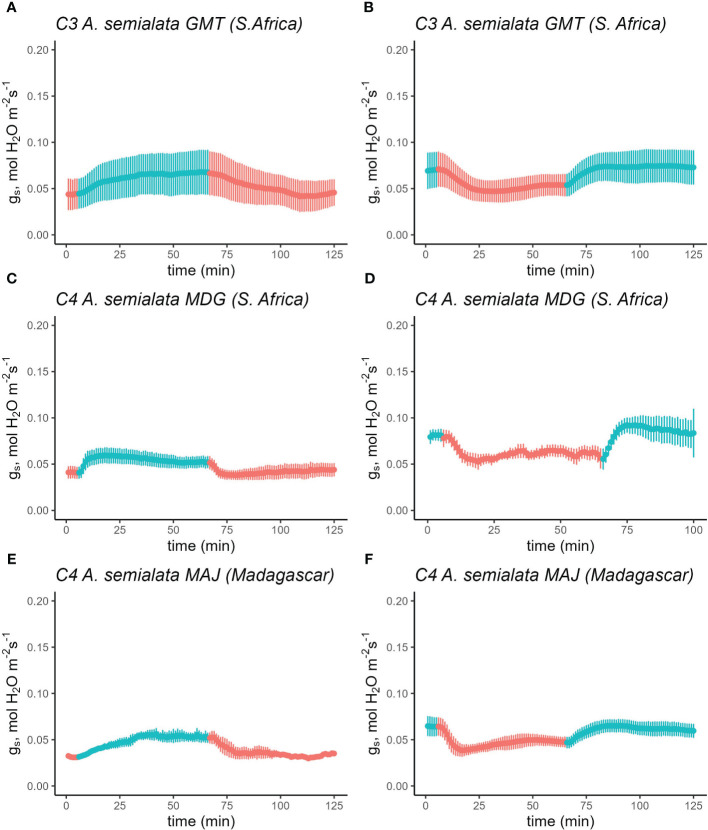
Time course of stomatal conductance (g_s_) response in C_3_ *A. semialata* subsp. eckloniana ‘GMT’ **(A, B)**, C_4_ *A. semialata* subsp. semialata ‘MDG’ **(C, D)** and C_4_ *A. semialata* subsp. semialata ‘MAJ’ **(E, F)** in response to a sequence of 75% red + 25% blue light and vice versa. Leaves were initially acclimated in either 100% red **(A, C, E)** or 75% red+25% blue light **(B, D, F)** until steady-state was achieved (time = 0 in the figure). Subsequently, the light environment was switched depending on the initial light condition while maintaining a photosynthetic photon flux density of 500 µmol m^-2^ s^-1^. The leaves were acclimated to the new light condition for 1 h before returning it back to the original light condition. Reference CO_2_ was maintained at 410 µmol mol^-1^, block temperature was kept at 25°C and average VPD was 1.2 kPa. Data points represent mean ± se (n=3-4).

Cleomaceae and *Flaveria* are both dicotyledonous species, but C_4_ photosynthesis is also quite prevalent in the monocots (Sage, 2004). Monocot species have stomatal features distinctly different from dicots (Bertolino et al., 2019). To determine if the stomatal responses described above also extend to congeneric C_3_ and C_4_ species in the monocots, the R*→*RB*→*R switchover experiment was also performed on C_3_ and C_4_ *A. semialata* subspecies (**Figure 3**). In this case, significant stimulation of g_s_ in response to the R*→*RB switch was observed across both C_3_ and C_4_ subspecies, increasing from 0.05 to 0.07 mol H_2_O m^-2^ s^-1^ and returning to 0.05 mol H_2_O m^-2^ s^-1^ in response to the RB*→*R switch.

After reaching steady state in R, g_s_ increased significantly from 0.05 mol H_2_O m^-2^ s^-1^ to 0.07 mol H_2_O m^-2^ s^-1^ in C_3_ *A. semialata* GMT upon the switch to RB, and then returned to 0.05 mol H_2_O m^-2^ s^-1^ when the light was shifted back to R (**Figure 3A**; **Table S9**). Both C_4_ *A. semialata* (‘MDG’ and ‘MAJ’) increased from 0.04 mol H_2_O m^-2^ s^-1^ to 0.05 mol H_2_O m^-2^ s^-1^ under RB, and back to 0.04 when the light was reversed to R (**Figures 3C, D**).

In the reverse sequence, g_s_ followed a similar pattern. In C_3_ *A. semialata* ‘GMT’, g_s_ was 0.07 mol H_2_O m^-2^ s^-1^ at the start of R, which then dropped to 0.05 mol H_2_O m^-2^ s^-1^ under R, and returned to 0.07 mol H_2_O m^-2^ s^-1^ when light was changed to RB (**Figure 3B**; **Table S10**). Following steady state, the initial g_s_ in C_4_

*A. semialata* ‘MDG’ under RB was 0.08 mol H_2_O m^-2^ s^-1^, which rapidly declined to 0.06 mol H_2_O m^-2^ s^-1^, with a final g_s_ of 0.06 mol H_2_O m^-2^ s^-1^ (**Figure 3D**). Similarly, g_s_ in C_4_ *A. semialata* ‘MAJ’ went from 0.07 to 0.05 and back to 0.07 mol H_2_O m^-2^ s^-1^ throughout of the sequence (**Figure 3E**).

Under R*→*RB*→*R and the reverse sequence, RB*→*R*→*RB, fitting the g_s_ data to a linear mixed-effects model (**Tables S8** and **S9**) showed a highly significant effect of light (R-RB-R, p=0.0007;RB-R-RB, p=0.0060), but the main effect of species (R-RB-R, p=0.3455; RB-R-RB, p=0.8859) and its interaction with light (R-RB-R, p=0.2055; RB-R-RB, p=0.7486) were not significantly different.

Although neither sequence had a clear effect on *A* (**Figure S4**), linear mixed-effects model analysis of *A* under R*→*RB*→*R showed a significant main effect of light (p=0.0013) (**Table S10**), this same relationship could not be detected in the reverse sequence (**Table S11**, p=0.1395). Both main effects in the reverse sequence were also not significant.

### Red light response in phylogenetically-close pairs of C_3_ and C_4_ at constant C_i_

To evaluate the quantitative red-light stomatal response, gas exchange was measured at different red PPFDs. The depletion of CO_2_ in the mesophyll can give rise to an apparent red light response (Roelfsema et al., 2002) which was not the focus of the experiments here, therefore the stomatal response to red light was evaluated under constant C_i_.

**Figure 4:**
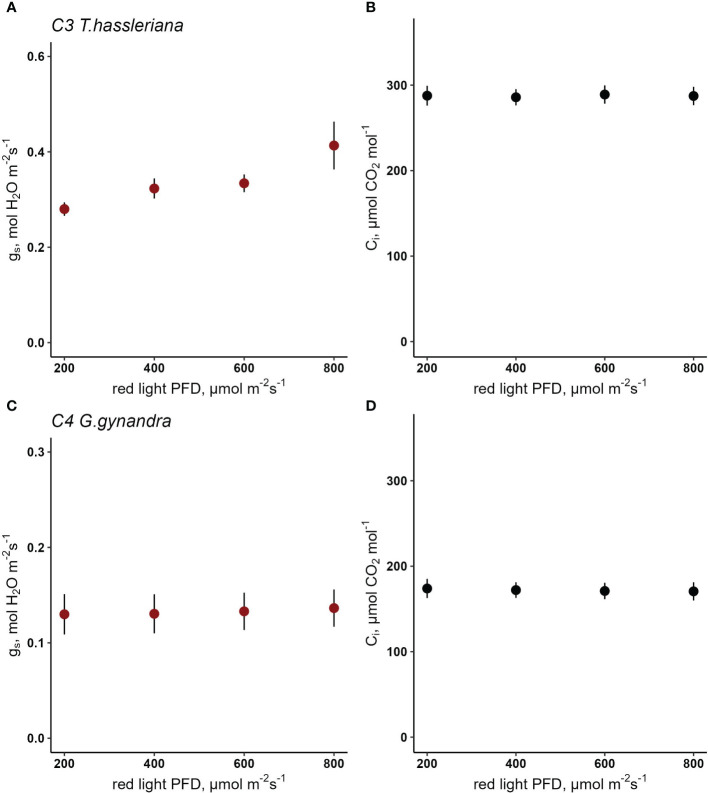
Response of stomatal conductance (g_s_) to red light photosynthetic photon flux density (PPFD) in C_3_ *T. hassleriana* **(A)** and C_4_ *G. gynandra* **(B)** at constant C_i_ **(C, D)**. A linear mixed- effects model analysis was carried out to test if photosynthetic type influences the response to red light in congeneric species belonging to Cleomaceae. Data points represent mean ± se (n=3-4).

In Cleomaceae, C_3_ *T. hassleriana* showed a drop in steady state g_s_ from 0.42 mol H_2_O m^-2^ s^-1^ to 0.26 mol H_2_O m^-2^ s^-1^ when red light intensity was reduced step-wise from 800 µmol m^-2^ s^-1^ to 200 µmol red photons m^-2^ s^-1^ (**Figure 4A**) while C_i_ was maintained at 288 µmol CO_2_ mol^-1^(**Figure 4B**). Stomatal conductance in C_4_ *G. gynandra* remained consistent around 0.15 mol H_2_O m^-2^ s^-1^ at the starting light intensity of 800 µmol m^-2^ s^-1^ red light (**Figure 4C**), while C_i_ was held constant at 173 µmol CO_2_ mol^-1^ (**Figure 4D**) and only slightly decreased to 0.12 mol H_2_O m^-2^ s^-1^ when light was set to 200 µmol red photons m^-2^ s^-1^. Using a linear mixed-effects model, the interaction of photosynthetic type and red PPFD on g_s_ was found to be significant (p=0.046) (**Table S14**), as a result of the significantly weaker red-light response in *G. gynandra* compared to *T. hassleriana*. In both species *A* significantly responded to red light PPFD (p*<*0.001)(**Figure S5** and **Table S15**).

**Figure 5:**
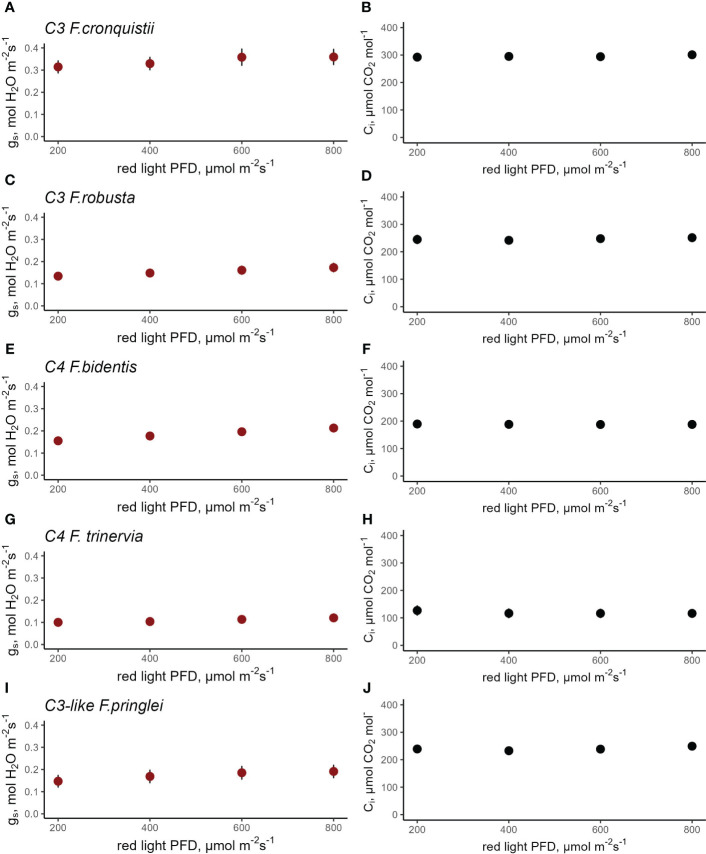
Response of stomatal conductance (g_s_) to red light photosynthetic photon flux density (PPFD) at constant intercellular CO_2_ concentration (C_i_) in C_3_ *F. cronquistii* (**A**, **B**, n=4) and C_3_ *F. robusta* (**C**, **D,** n=5), C_4_ *F. bidentis* (**E**, **F**, n=8), C_4_ *F. trinervia* (**G**, **H**, n=5), and C_3_-like *F. pringlei* (**I**, **J,** n=5). A linear mixed- effects model analysis was carried out to test if photosynthetic type influences the response to red light in congeneric species belonging to *Flaveria*. Data points represent the mean ± se.

In *Flaveria*, C_3_ *F. cronquistii* showed an average *g*_s_ of 0.36 mol H_2_O m^-2^ s^-1^ at 800 μmol red photons m^-2^ s^-1^ which decreased to 0.27 mol H_2_O m^-2^ s^-1^ at 200 μmol photons m^-2^ s^-1^ while C_i_ was kept constant at 296 μmol CO_2_ mol^-1^ (**Figures 5A, B**). C_3_ *F. robusta* had a lower overall g_s_, which started from 0.17 mol H_2_O m^-2^ s^-1^ and decreased to 0.13 mol H_2_O m^-2^ s^-1^ at a C_i_ of 247 mol CO_2_ mol^-1^ (**Figures 5C, D**). Meanwhile steady state g_s_ in C_4_ *F. bidentis* under 800 μmol red photons m^-2^ s^-1^ was 0.18 mol H_2_O m^-2^ s^-1^ which slowly decreased to 0.13 mol H_2_O m^-2^ s^-1^with the decrease in light intensity (**Figure 5E**), but while holding C_i_ constant at 157 μmol CO_2_ mol^-1^ (**Figure 5F**). g_s_ in C_4_ *F. trinervia* was practically unchanged in response to red light intensity ranging between 0.12 and 0.10 H_2_O m^-2^ s^-1^(**Figures 5G, H**). Finally, C_3_-like *F. pringlei* had a g_s_ of 0.19 mol H_2_O m^-2^ s^-1^ to 0.15 mol H_2_O m^-2^ s^-1^ (**Figure 5I**) as light intensity was reduced while keeping C_i_ at 240 μmol CO_2_ mol_-1_ (**Figure 5J**). Fitting the g_s_ data to a linear mixed-effects model showed a highly significant species (p<0.0001) and red-light PPFD (p<0.0001) main effects, and a non-significant interaction effect (p=0.6789) (**Table S15**). The significance of the red-light effect demonstrates that despite the limited range of the response, stomata did respond significantly to red-light. The strong significance of the species effect demonstrates that the absolute values of stomatal conductance differed significantly between *Flaveria* species in line with photosynthetic type. However, the presence of a non-significant interaction between species and red light demonstrates that the magnitude of the stomatal red-light response did not differ significantly between these species. Not surprisingly, CO_2_ assimilation rate responded strongly to light intensity in all five species (**Figure S4** and **Table S16**), suggesting that the significant stomatal red-light response in these species may serve to fine-tune coordination between stomatal conductance and photosynthesis, in concert with the stomatal CO_2_ response.

**Figure 6:**
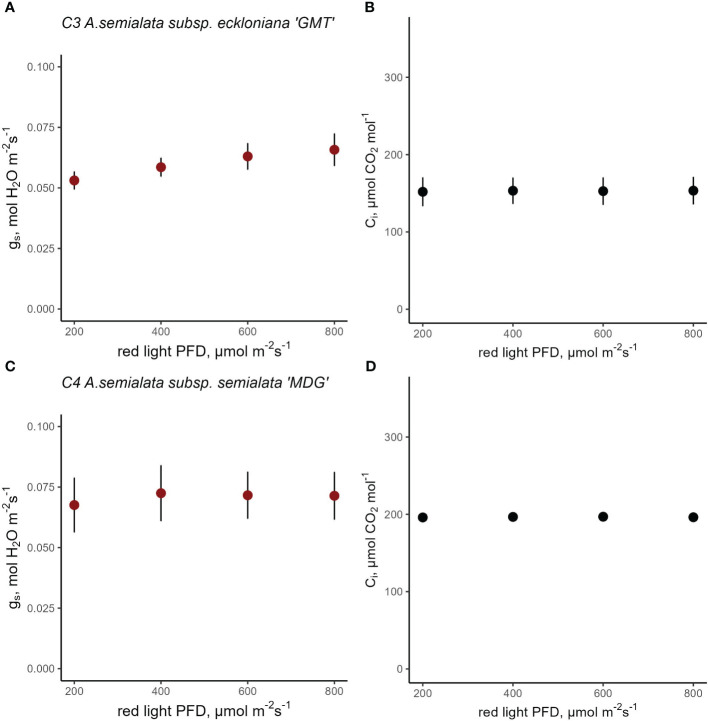
Response of stomatal conductance (g_s_) at constant intercellular CO_2_ concentration (C_i_) in C_3_ *A. semialata* subsp. eckloniana ‘GMT’ (**A**, **B**, n=4) and C_4_ *A. semialata* subsp.semialata ‘MDG’ (**C**, **D**, n=5). Linear mixed-effects model analysis was carried out to test if photosynthetic type influences the response to red light in C_3_ and C_4_ subspecies belonging to *Alloteropsis*. Each data point represents the mean ± se.

The stomatal red-light response was also characterized for C_3_ *A. semialata* subsp. eckloniana and C_4_ *A. semialata* subsp. semialata (**Figures 6A, C**). When PPFD was reduced from 800 to 200 µmol red photons m^-2^ s^-1^ for C_3_ *A. semialata* subsp. eckloniana, g_s_ changed very little from 0.07 to 0.05 mol H_2_O m^-2^ s^-1^ (**Figures 6A, B**). Similarly, the C_4_ *A. semialata* subsp. semialata kept g_s_ at approximately 0.07 mol H_2_O m^-2^ s^-1^ despite the decrease in red PPFD (**Figures 6C, D**). Curiously the C_4_ subspecies (**Figure 6D**) had a higher C_i_ than the C_3_ subspecies at 800 µmol red photons m^-2^ s^-1^ (**Figure 6C**; 153 vs 193 µmol CO_2_ mol^-1^). A mixed-effects model detected a marginal effect of red PPFD p=0.0525) but there was no evidence of the effect of subspecies (p=0.3884) and its interaction with red PPFD (**Table S17**, p=0.1979). Similar to Cleomaceae and *Flaveria*, assimilation rate also responded strongly to red-light PPFD (**Figure S7** and **Table S18**).

Altogether, these data established a significant stomatal red light opening response to red-xlight, independent of C_i_ in Cleomaceae and most *Flaveria* species, but not in *Alloteropsis*. Although the observed reduced stomatal sensitivity to red light in C_4_ *G. gynandra* compared to C_3_ *T. hassleriana* is consistent with the hypothesized differences between C_3_ and C_4_ photosynthetic types, the differences observed between *Flaveria* species and the lack of these in *Alloteropsis* seem to suggest that these are more likely to be species-specific responses, rather than generic differences between photosynthetic pathways.

### Stomatal morphology and density

**Figure 7:**
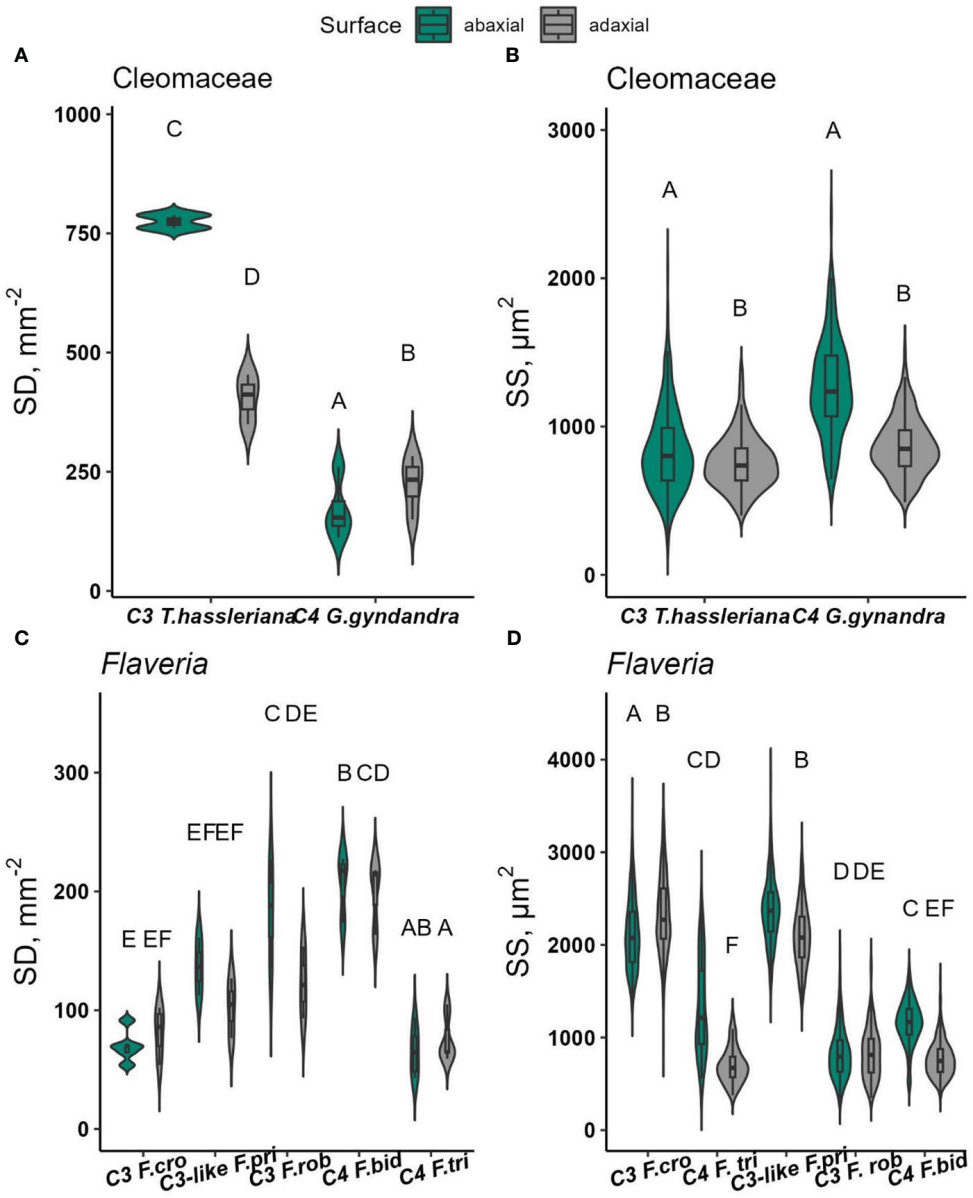
Stomatal density (SD, stomata mm^-2^) and stomatal size (SS, *µ*m^2^) on the abaxial and adaxial leaf surfaces of Cleomaceae **(A, B)**, *Flaveria* **(C, D)**. Data were collected from 1-4 random microscopic fields of view from abaxial and adaxial surfaces from 3-5 biological replicates. In each species, at least 350 stomata per surface were measured for SS, with the exception of C_3._ *F. cronquistii* and C_4_ *F. trinervia*, for which at least 141 and 170 stomata per surface were measured, respectively. Violin plots with embedded box plots in the same panel, with the same letters are not significantly different at *α* = 0.05 using Tukey’s HSD test.

Stomatal density and size were determined in conjunction with the gas exchange measurements described in the previous sections. In Cleomaceae, the overall stomatal density was significantly higher in C_3_ *T. hassleriana* (590 stomata mm^-2^) than in C_4_ *G.gynandra* (200 stomata mm^-2^) (p*<*0.0001). The distribution of stomata on both leaf surfaces was also found to be significantly different, but in a species-dependent manner (**Figure 7A**, p=*<*0.0001). The SD on the adaxial surface was almost two times higher than the abaxial surface in C_3_ *T. hassleriana* (**Figures S8A, B**). In contrast, in C_4_ *G.gynandra*, SD was 25% higher on the adaxial compared to the abaxial surface (**Figures S8C, D**).

While density varied depending on leaf surface in Cleomaceae, stomatal size was significantly larger on the abaxial than the adaxial surface (Leaf surface, p <0.0001) (**Figure S9A**). This SS difference was similar in both Cleomaceae species.

SD among *Flaveria* species (**Figures 7C** and **S9A-J**) were significantly different (p*<*0.0001), but the trend in SD was not consistent with the proposed progression of larger and fewer stomata in C_4_ *Flaveria* than in C_3_ *Flaveria* (Zhao et al., 2022). Instead, *F. cronquistii*, one of the two C_3_ species, had the lowest SD and the highest SD was found for *F. bidentis*, one of the C_4_ species. Thus, these differences do appear to be species-dependent, but not determined by photosynthetic pathway. SD was similar between both leaf surfaces in both C_4_ *Flaveria* species (**Figure S9C**) as well as in the C_3_ *F. cronquistii*, whereas the abaxial surface had higher SD in the C_3_-like *F. pringlei* and the C_3_ *F. robusta* (significant for the latter, p*<*0.05). SS was higher on the abaxial side for all *Flaveria* species except for C_3_ *F. cronquistii* resulting in an interaction effect of species x leaf surface which was highly significant (p*<*0.0001, **Figure S7D**).

Meanwhile, there was no statistically significant difference in SD detected between the *Alloteropsis* subspecies (p = 0.338) (**Figures 7** and **S10**), while SS was larger on the abaxial surface (p <0.05, **Figure 7F**). Altogether, stomatal density, distribution and anatomical traits appeared to be largely determined by species effect, rather than photosynthetic pathway, although notably, all C_4_ species had at least equal SD on the adaxial and abaxial surfaces, whereas the more commonly found bias of SD towards the abaxial surface was only found in some of the C_3_ species.

## Discussion

Leaf-level g_s_ responses to red light quantity or red/blue spectral composition were compared between C_3_ and C_4_ species from Cleomaceae, *Flaveria*, and *Alloteropsis* genera. All three groups are well-studied models in the quest to understand evolution of C_4_ photosynthesis. Combining experimental comparisons within all three genera allowed for a global comparison of stomatal light responses between C_3_ and C_4_ photosynthetic types while controlling for the effects of phylogenetic distance within each genus.

### C_4_ species from the genus *Cleomaceae* and *Flaveria* are less sensitive to blue light than the C_3_ counterparts

BL promotes diverse physiological plant responses ranging from phototropism, chloroplast photorelocation movement, leaf flattening, leaf positioning, and stomatal opening (Christie, 2007; Inoue et al., 2008; Goh, 2009). Blue-light induced stomatal opening is probably the most well-characterized among these responses. However, the stomatal opening response to BL is not universal. Studies in model and non-model species provide strong evidence of the species-specificity of the BL stomatal opening response (Vialet-Chabrand et al., 2021). Differences in BL-dependent stomatal opening may exist. A recent study reported that C_4_ crop species (Zhen and Bugbee, 2020) displayed diminished stomatal opening to BL compared to C_3_ crops. Indeed, in this study, C_4_ dicots were found to be less sensitive to blue light-induced stomatal opening than close relatives operating the C_3_ pathway. This observation demonstrates a clear difference between C_3_ and C_4_ opening response, as hypothesized. In contrast, both the C_3_ and C_4_ accessions from the monocot *A. semialata* displayed blue light-dependent stomatal opening, which meant that the C_4_ photosynthetic pathway *per se* does not determine this reduced blue light sensitivity.

The decreased sensitivity of C_4_ dicots to blue light may be a result of decreased guard cell expression of phototropins, the major blue light photoreceptor in plants. Analysis of transcript abundance in different cell types of C_4_ *G. gynandra* showed that the transcript abundance of PHOT1and PHOT2, as well as another blue light photoreceptor, CRY2, were lower in mesophyll and guard cells of C_4_ *G. gynandra*, than in C_3_

*T. hassleriana* Aubry et al. (2016). In *Arabidopsis*, phototropins are expressed in almost every plant part Sakamoto and Briggs (2002), however the strongest expression in the epidermis is detected in guard cells, where phots are associated with the plasma membrane ((Sakamoto and Briggs, 2002)). PHOT1 and 2 have partially overlapping roles. PHOT1 has been suggested to respond to lower PPFD (0.1-50 µmol m^-2^ s^-1^), while PHOT2 responds to PPFD up to 250 µmol m^-2^ s^-1^ in *Arabidopsis* [15]. Several hypotheses exist with regards to the function of the stomatal blue light response. One putative role is to stimulate photosynthesis via enhanced stomatal opening in the morning hours when blue light is more prevalent. In the results presented here, PPFD was kept equal during the spectral changes, and CO_2_ assimilation rate was invariable, despite significant changes in stomatal conductance in C_3_ *T. hassleriana*, C_3_-like *F. pringlei*, C_3_ *F. robusta*, C_3_ *F. cronquistii* and both *A. semialata* subspecies. These observations do not seem consistent with an important role for blue light induced stomatal opening for photosynthetic carbon gain.

Could there be other benefits to blue sensitivity of stomatal movements? An alternative hypothesis to explain the differential sensitivity to blue light could be related to leaf thermoregulation. Stomatal opening in response to blue light has been suggested to work as a proxy for high-intensity sunlight (Zhao et al., 2022) and function primarily to cool the leaf via transpiration (Taylor et al., 2012). Furthermore, photorespiration increases with temperature in C_3_ species but does not change in C_4_ species due to CCM (Lawson et al., 2008; Arrivault et al., 2017). Hence, net CO_2_ assimilation rate in C_4_ species has a higher optimum temperature than in C_3_ species (Xu et al., 2016), and a slight elevation of leaf temperature can substantially increase the photosynthesis rate in C_4_, whereas the temperature response between 20°C and 30°C in C_3_ species is almost negligible. Based on these differences, the presence of stomatal blue light response may help to cool the leaves of the C_3_ dicot species studied here, and thus keep photorespiration lower, while the lack of the stomatal blue light response in the C_4_ dicots may help elevate leaf temperature and thereby stimulate CO_2_ assimilation rate.

Interestingly, this would also be consistent with the putative involvement of diminished PHOT expression, since PHOTs also perform thermosensory roles Fujii et al. (2017) and promote evapotranspiration and leaf cooling at high temperatures in *Arabidopsis* (Kostaki et al., 2020).

### Differences in stomatal red-light response are determined by species, rather than photosynthetic pathway

The mechanism underlying the red-light response remains unresolved. Unlike the blue light response, red light-induced stomatal opening does not seem to involve direct signal transduction from red light photoreceptors in the guard cells but rather relies on the decrease in C_i_ through the consumption of CO_2_ via mesophyll photosynthesis (Mott, 1988; Roelfsema et al., 2002; Lawson et al., 2008). However, g_s_ was also observed to increase with light despite constant or high C_i_ or after achieving steady-state photosynthesis (Matrosova et al., 2015), which implies other signals than C_i_ could also be involved (Lawson et al., 2014; Flu¨tsch and Santelia, 2021).

C_4_ photosynthesis operates in a two-cell compartment system, such that a strong metabolite gradient is essential to run it efficiently (Leegood and von Caemmerer, 1988; Arrivault et al., 2017). Precise coordination between the C_3_ and C_4_ cycles is crucial under varying irradiances and C_i_ to avoid excessive CO_2_ leakage from the bundle sheath (Kromdijk et al., 2014). C_3_ photosynthesis, on the other hand, is less complex. We therefore hypothesized that species with these contrasting mesophyll photosynthesis characteristics may also be expected to show a different stomatal response to red light.

However, the comparison between the red-light response in C_3_ and C_4_ dicot species showed that although significant variation in the stomatal red-light response was found between species, no structural differences between C_3_ and C_4_ species were found across the three phylogenetically controlled comparisons. Instead, a distinct red-light response was found in C_3_ *T. hassleriana*, and a weaker, but significant red-light effect was also found across all *Flaveria* species, suggesting that these species support the coordination between stomatal conductance and photosynthesis via stomatal responses to both C_i_ and red light. In contrast, C_4_ *G. gynandra* as well as both *A. semialata* subspecies appear to rely solely on the C_i_ response.

One aspect of the red-light response debate is whether stomatal guard cells can independently respond to red light. Indeed, a recent metabolomics study reported a direct guard cell response to red light (Zhu et al., 2020). This finding could be consistent with a putative mechanism involving the phosphorylation of the guard cell plasma membrane H^+^ATPase leading to stomatal opening in intact leaves (Ando and Kinoshita, 2018). Consistent with this mechanism, it was demonstrated that H^+^ATPase activation by red light was dependent on fluence rate (Ando and Kinoshita, 2019). If this mechanism can be shown to play a significant role, it may offer an alternative explanation for the observed species differences in the red light response, not necessarily dependent on a mesophyll-derived signal.

### Role of stomatal morphology and density on stomatal movements in response to light

**Figure 8:**
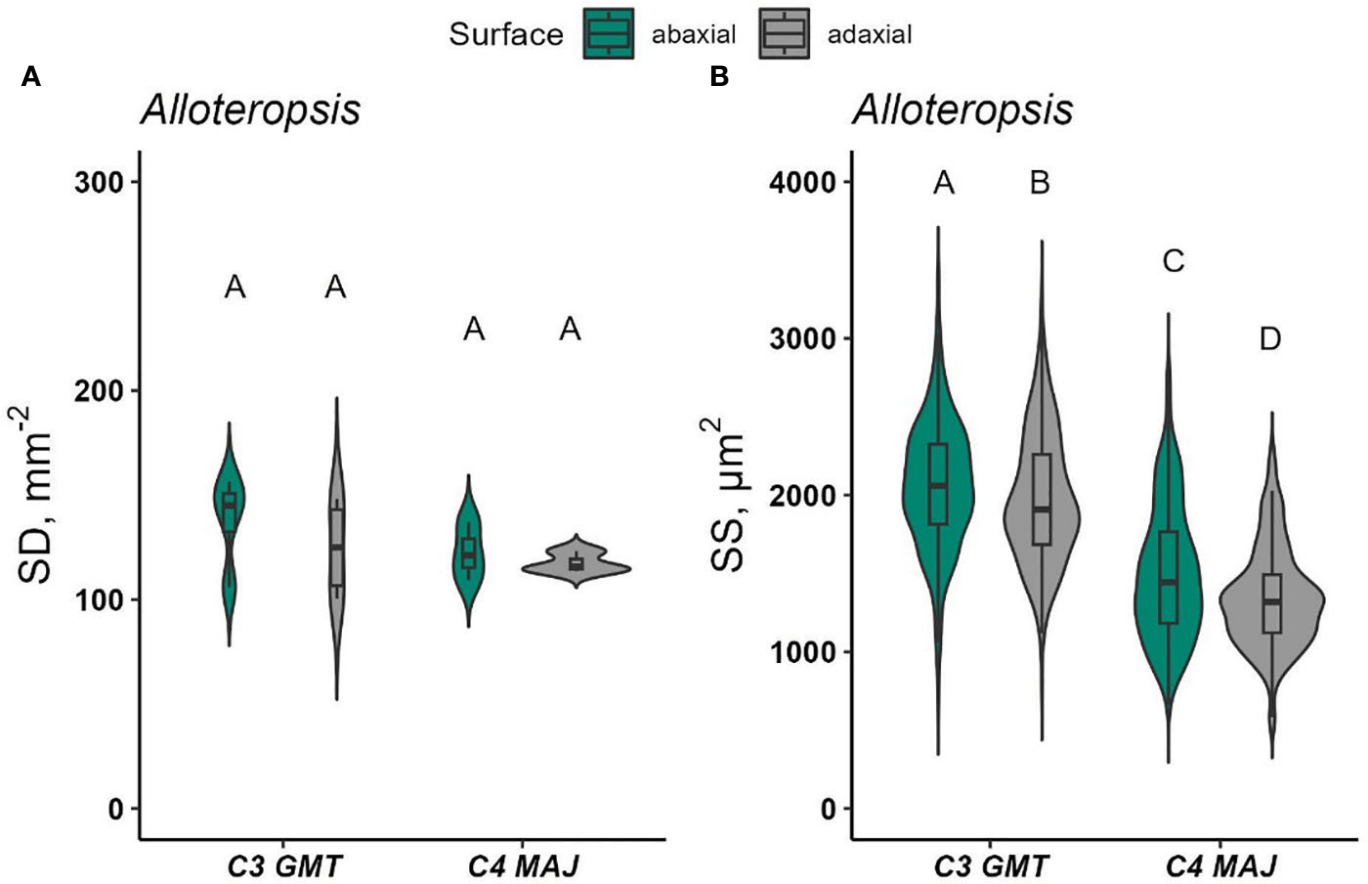
Stomatal density (SD, stomata mm^-2^) and stomatal size (SS, *µ*m^2^) on the abaxial and adaxial leaf surfaces of *Alloteropsis* **(A, B)**. Data were collected from 1-4 random microscopic fields of view from abaxial and adaxial surfaces from 3-5 biological replicates. In each species, at least 350 stomata per surface were measured for SS. Violin plots with embedded box plots in the same panel, with the same letters are not significantly different at *α* = 0.05 using Tukey’s HSD test.

Stomatal density and morphology are well-known to impact responses to light (Harrison et al., 2020). These traits varied significantly between species studied here (**Figures 7**, **8**). For most dicots, such as Cleomaceae and *Flaveria*, stomata are defined by a pair of kidney-shaped guard cells (**Figures S8 and S9**). In contrast, stomata in the monocot *A. semialata* subspecies are dumbbell-shaped with additional subsidiary cells flanking the guard cells (**Figure S10**). The morphology and size of stomata in the grasses are often suggested to facilitate more rapid responses to short-term environmental perturbations (Chen et al., 2017), such as fluctuating light or acute changes to temperature or VPD (Bauer et al., 2013). However, there was no evidence in this study linking stomatal morphology nor size to blue light-induced opening or quantitative red-light responses.

Within the dicots, C_3_ *T. hassleriana* (Cleomaceae) had denser and smaller stomata than the C_4_ species *G. gynandra*, which was consistent with earlier observations Aubry et al. (2016). The higher SD on the adaxial surface could be responsible for the pronounced red-light response, independent of C_i_ in C_3_ *T. hassleriana*. However, measurements on intact leaves do not make it possible to derive whether these responses rely on signal perception in the guard cell, or in the underlying mesophyll.

Furthermore, in *Flaveria*, SD and SS varied between species. Unlike in the Cleomaceae species, SD or SS had no evident relationship to the red-light response in *Flaveria*. Additionally, the observed between-species variability in SD and SS in *Flaveria* was inconsistent with the proposed trajectory of stomatal density and guard cell length (and therefore, size) changes during C_4_ evolution, where C_3_ *Flaveria* species tended to have smaller but more stomata, while C_4_ *Flaveria* show increased stomatal size and decreased density (Zhao et al., 2022). In fact, C_3_-like *F. pringlei* and C_3_ *F. cronquistii*, were found to have larger stomata (**Figure 7D**), whereas both C_4_ *Flaveria* had smaller stomatal sizes. The discrepancy between this study and Zhao et al. (2022) might have stemmed from very low sample sizes used by the latter. Stomatal parameters in Zhao et al. (2022) were measured from a minimum of 5 individual stoma, to at most 10 stomata per species, which, as shown in **Figure 7D**, is insufficient to capture size differences among *Flaveria* species.

Another possible reason for these discrepancies is the potential impact of ecological adaptation in some of the *Flaveria* species. C_3_ *F. cronquistii* had equal stomatal distribution on both leaf surfaces like the C_4_ *Flaveria* species, but with twice larger stomatal size on either leaf surface. In terms of leaf shape, C_3_ *F. cronquistii* has linear, elongated leaves, whereas the other *Flaveria* representatives display elliptic or ovate leaf shapes. At an ecological standpoint, narrower leaves such as those in C_3_ *F. cronquistii* perhaps reflect an adaptation in minimizing excessive heat load (Leigh et al., 2017) whilst maximizing net carbon gain (Michaletz et al., 2016) and together with a uniform stomatal distribution and a larger proportion of large stomata allow it to sustain higher g_s_ rates to effect cooling.

## Conclusion

Here we used three phylogenetically-controlled comparisons to assess differences between stomatal anatomy and stomatal responses to red and blue light. The C_4_ species from the genus *Cleomaceae* and *Flaveria* in this study did not have a detectable blue light stomatal response, unlike their C_3_ counterparts. However, perhaps surprisingly, the results demonstrate that the impact of photosynthetic pathway and stomatal morphology and distribution were not as strong as initially hypothesized, but instead varied between genera. Similarly, the quantitative red-light response showed significant species variation but no association with photosynthetic pathway. Altogether, the findings suggest that the evolution of C_4_ photosynthesis in the dicots may have led to a change in light-regulated stomatal movements, challenge the general nature of previously observed stomatal morphological differences between C_3_ and C_4_ species (Zhao et al., 2022) and demonstrate the importance of controlling for evolutionary distance.

## Data availability statement

The original contributions presented in the study are included in the article/**Supplementary Material**. Further inquiries can be directed to the corresponding author.

## Author contributions

EB: Writing – original draft; Experimentation and analysis. CS: Writing – review & editing. LC: Writing – review & editing. RV: Writing – review & editing. JK: Conceptualization, Writing – review & editing.
