# Supplementary material for: A comparison of stomatal conductance responses to blue and red light between C3 and C4 photosynthetic species in three phylogenetically-controlled experiments
Source: Front Plant Sci. 2023 Sep 27;14:1253976. doi: 10.3389/fpls.2023.1253976 (PMC10565490; doi:10.3389/fpls.2023.1253976)
Supplement: Supplementary file 1 [file DataSheet_1.pdf]

Supplementary Material

A comparison of stomatal conductance responses to blue and red light between C_3 and C_4 photosynthetic species in three phylogenetically-controlled experiments

Emmanuel L. Bernardo [1,2], Cristina Rodrigues Gabriel Sales [1], Lucía Arce Cubas [1], Richard L. Vath [1] and Johannes Kromdijk [1,3,*]

[1]*Department of Plant Sciences, University of Cambridge, Downing Street, CB2 3EA Cambridge, United Kingdom.*
[2]*Institute of Crop Science, College of Agriculture and Food Science, University of the Philippines Los Baños, College, Laguna 4031, The Philippines.*
[3]*Carl R. Woese Institute for Genomic Biology, University of Illinois at Urbana-Champaign, 61801 Urbana, Illinois, USA.*

Correspondence*:
Johannes Kromdijk
jk417@cam.ac.uk

Supplementary Tables

Table S1. Repeated measures (mixed-effects) ANOVA of g_s in Cleomeaceae under R→RB→R sequence (R^2=0.96, RMSE = 0.044).

Source	DF	F ratio	Pr(>F)
Species	1	85.88	0.0002
Light	2	8.90	0.0060
Species x Light	2	8.10	0.0082

Table S2. Repeated measures (mixed-effects) ANOVA of g_s in Cleomeaceae under RB→R→RB sequence (R^2=0.93, RMSE = 0.029).

Source	DF	F ratio	Pr(>F)
Species	1	16.99	0.0092
Light	2	14.45	0.0011
Species x Light	2	12.52	0.0019

Table S3. Repeated measures (mixed-effects) ANOVA of *A* in Cleomeaceae underR→RB→R sequence (R^2=0.98, RMSE = 0.397).

Source	DF	F ratio	Pr(>F)
Species	1	24.43	0.0043
Light	2	7.379	0.0108
Species x Light	2	0.9126	0.4325

Table S4. Repeated measures (mixed-effects) ANOVA of *A* in Cleomeaceae under RB→R→RB sequence (R^2=0.93, RMSE = 0.87).

Source	DF	F ratio	Pr(>F)
Species	1	1.519	0.2832
Light	2	2.275	0.1395
Species x Light	2	1.167	0.3792

Table S5. Repeated measures (mixed-effects) ANOVA of g_s in three species of *Flaveria* under R→RB→R sequence (R^2=0.97, RMSE = 0.017).

Source	DF	F ratio	Pr(>F)
Species	2	711.862	<0.0001
Light	2	12.635	0.0011
Species x Light	4	11.340	0.0005

Table S6. Repeated measures (mixed-effects) ANOVA of *A* in three species of *Flaveria* under RB→R→RB sequence (R^2=0.99, RMSE = 0.014)

Source	DF	F ratio	Pr(>F)
Species	2	4.22	0.0717
Light	2	3.793	0.0529
Species x Light	4	2.2190	0.1285

Table S7. Repeated measures (mixed-effects) ANOVA of g_s in five species of *Flaveria* under R→RB→R sequence (R^2=0.97, RMSE = 0.0017).

Source	DF	F ratio	Pr(>F)
Species	4	85.88	0.0002
Light	2	8.90	0.0001
Species x Light	8	8.08	0.0048

Table S8. Repeated measures (mixed-effects) ANOVA of *A* in five species of *Flaveria* under R→RB→R sequence (R^2=0.99, RMSE = 0.0481).

Source	DF	F ratio	Pr(>F)
Species	4	6.916	0.0015
Light	2	72.105	<0.0001
Species x Light	8	2.948	0.0122

Table S9. Repeated measures (mixed-effects) ANOVA of g_s in *Alloteropsis* under R→RB→R sequence (R^2=0.91, RMSE = 0.009).

Source	DF	F ratio	Pr(>F)
Species	2	0.252	0.7837
Light	2	12.72	0.0007
Species x Light	4	0.635	0.6457

Table S10. Repeated measures (mixed-effects) ANOVA of g_s in *Alloteropsis* under RB→R→RB sequence (R^2=0.87, RMSE = 0.011).

Source	DF	F ratio	Pr(>F)
Species	2	0.123	0.8859
Light	2	7.53	0.0060
Species x Light	4	0.482	0.7486

Table S11. Repeated measures (mixed-effects) ANOVA of *A* in *Alloteropsis* under R→RB→R sequence (R^2=0.98, RMSE = 0.510).

Source	DF	F ratio	Pr(>F)
Species	2	0.094	0.9117
Light	2	11.11	0.0013
Species x Light	4	1.968	0.1550

Table S12. Repeated measures (mixed-effects) ANOVA of *A* in *Alloteropsis* under RB→R→RB sequence (R^2=0.90, RMSE = 0.872).

Source	DF	F ratio	Pr(>F)
Species	2	1.519	0.2832
Light	2	2.273	0.1395
Species x Light	4	1.1378	0.3792

Table S13. Repeated measures (mixed-effects) ANOVA of *A* in Cleomaceae under varying intensities of red light (R^2=0.024, RMSE = 0.977).

Source	DF	F ratio	Pr(>F)
Species	1	45.21	0.0005
PPFD	1	10.09	0.0192
Species x PPFD	1	8.148	0.0291

Table S14. Repeated measures (mixed-effects) ANOVA of g_s in Cleomaceae under varying intensities of red light (R^2=1.825, RMSE = 0.93).

Source	DF	F ratio	Pr(>F)
Species	1	6.49	0.0437
PPFD	1	169.26	<0.0001
Species x PPFD	1	4.42	0.0802

Table S15. Repeated measures (mixed-effects) ANOVA of g_s in *Flaveria* under varying intensities of red light (R^2=0.027, RMSE = 0.91).

Source	DF	F ratio	Pr(>F)
Species	4	14.88	<0.0001
PPFD	1	59.608	<0.0001
Species x PPFD	1	0.506	0.6789

Table S16. Repeated measures (mixed-effects) ANOVA of *A* in *Flaveria* under varying intensities of red light (R^2=1.926, RMSE = 0.92).

Source	DF	F ratio	Pr(>F)
Species	4	10.37	0.0002
PPFD	1	516.72	<0.0001
Species x PPFD	1	11.42	<0.0001

Table S17. Repeated measures (mixed-effects) ANOVA of g_s in *Alloteropsis* under varying intensities of red light (R^2=0.99, RMSE = 0.002).

Source	DF	F ratio	Pr(>F)
Species	1	0.864	0.3884
PPFD	1	5.814	0.0525
Species x PPFD	1	2.095	0.1979

Table S18. Repeated measures (mixed-effects) ANOVA of *A* in *Alloteropsis* under varying intensities of red light (R^2=0.88, RMSE = 0.797).

Source	DF	F ratio	Pr(>F)
Species	1	0.136	0.7255
PPFD	1	309.08	<0.0001
Species x PPFD	1	10.353	0.0182

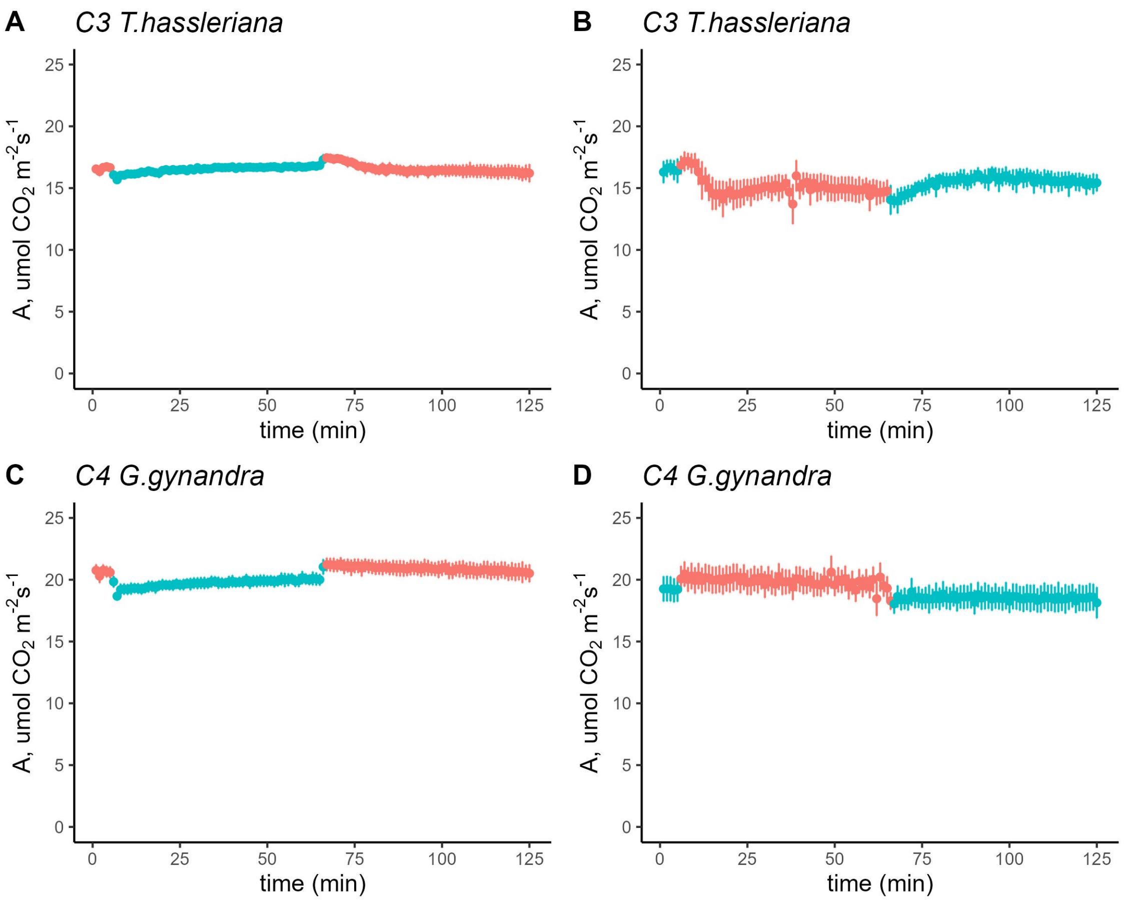

Figure S1. Time course of CO_2 assimilation (*A*) in C_3 *T. hassleriana* (**A** and **B**) and C_4 *G. gynandra* (**C** and **D**) in response to a sequence of 100% red to 75% red + 25% blue light and vice versa. Leaves were initially acclimated under either 100% red (**A** and **C**) or 75% red+25% blue light (**B** and **D**) until steady state was achieved. The light environment was switched depending on the initial light condition while maintaining a photosynthetic photon flux density of 500 µmol m^{-2} s^{-1}. The leaves were acclimated in the new light condition for 1 hour before returning it back to the original condition. Light conditions were changed at t_6 and t_{65}. Reference CO_2 was maintained at 400 µmol mol^{-1}, block temperature was kept at 25°C and average VPD was 1.2 kPa. Data points represent mean ± se (n = 3-4).

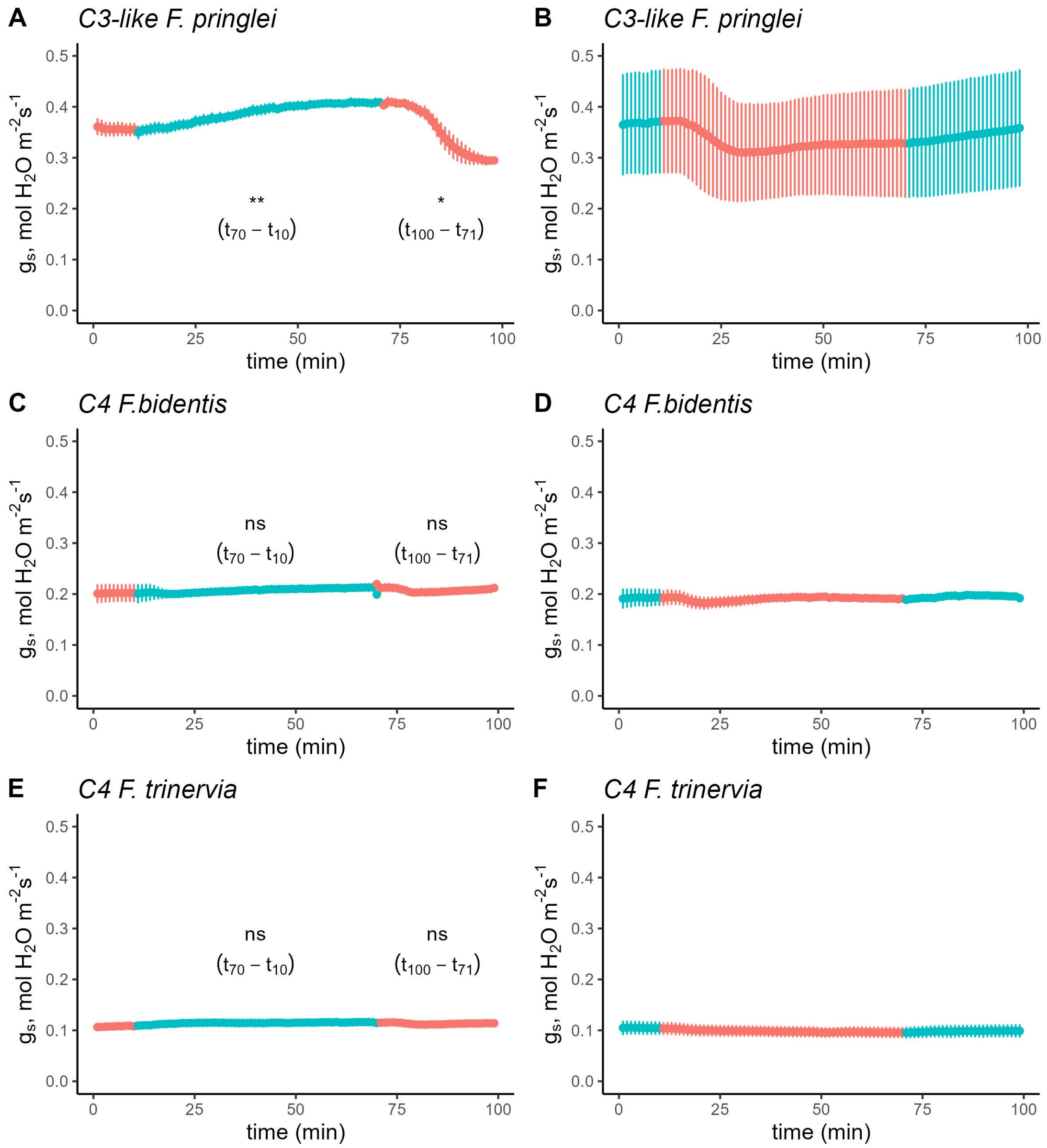

Figure S2. Time course of stomatal conductance (g_s) in C_3-like *F. pringlei* (**A** and **B**), C_4 *F. bidentis* (**C** and **D**), and C_4 *F. trinervia* (**E** and **F**) in response to a sequence of 75% red + 25% blue light and vice versa. Leaves were initially acclimated in either 100% red (**A**, **C**, and **E**) or 75% red+25% blue light (**B**, **D**, and **F**) until steady state was achieved. Subsequently, the light environment was switched depending on the initial light condition while maintaining a photosynthetic photon flux density of 500 µmol m^{-2} s^{-1}. The leaves were acclimated in the new light condition for 60 min before returning it back to the original condition for another 30 mins before terminating the experiment. Light conditions were reversed at t_{10} and t_{70}. Reference CO_2 was maintained at 400 µmol mol^{-1}, block temperature was kept at 25°C and average VPD was 1.2 kPa. Data points represent mean ± se (n=3).

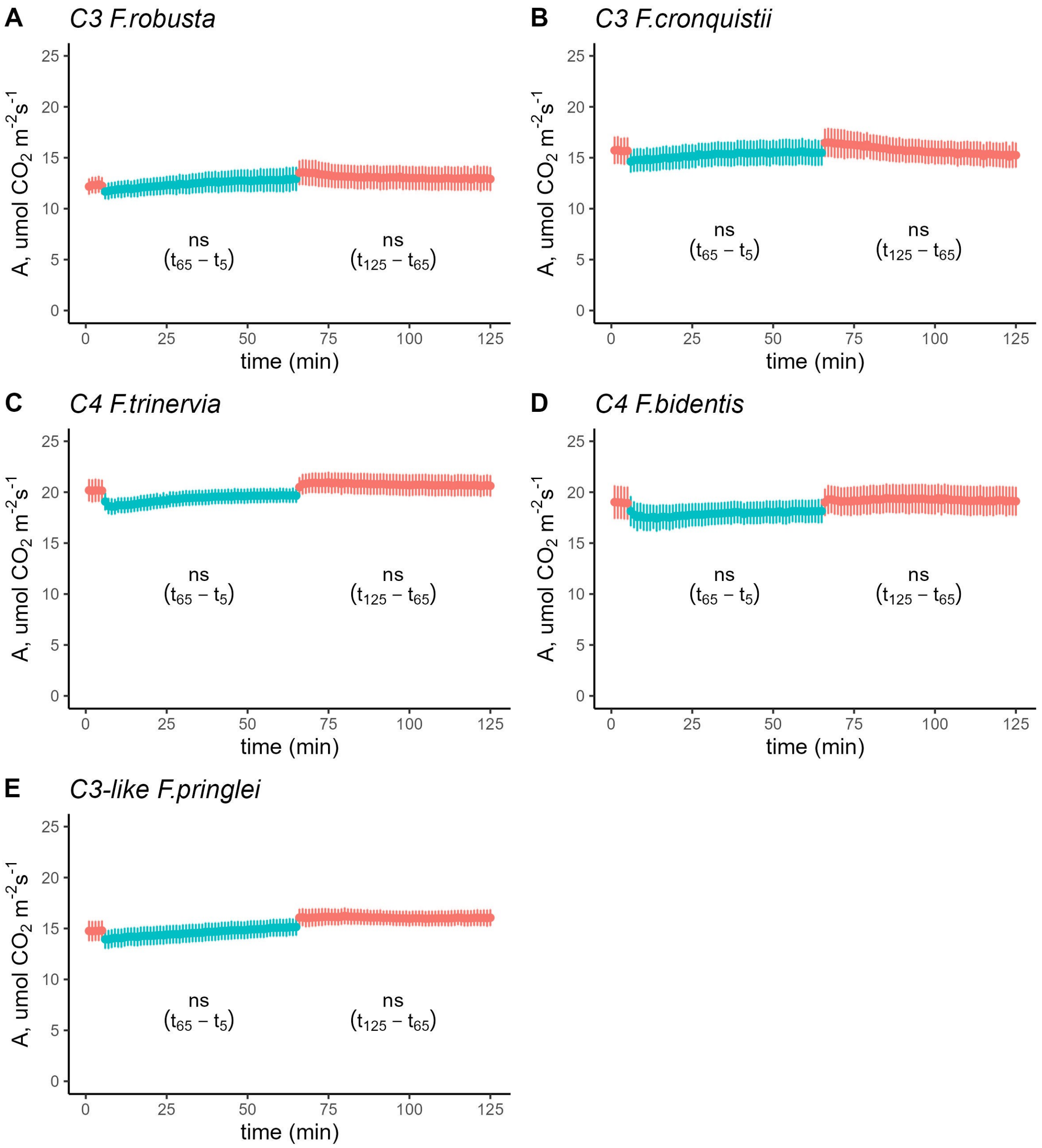

Figure S3. Time course of A in C_3 *F.robusta* (**A**), C_3 *F. cronquistii* (**B**), C_4 *F. trinervia* (**C**), C_4 *F. bidentis* (**D**), and C_3-like *F. pringlei* (**E**) in response to a sequence of 100% red and 75% red + 25% blue light. Leaves were initially acclimated under 100% red light until steady state was achieved. Subsequently, the light environment was switched to 75% red + 25% blue light while a photosynthetic photon flux density of 500 μmol m^{-2} s^{-1}. The leaves were acclimated to the new light condition for 60 min before returning them back to the original condition for another 60 mins before terminating the experiment. Light conditions were reversed at t_6 and t_{65}. Reference CO_2 was maintained at 410 μmol mol^{-1}, block temperature was kept at 25°C and average VPD was 1.2 kPa. Data points represent mean ± se (n=4-5).

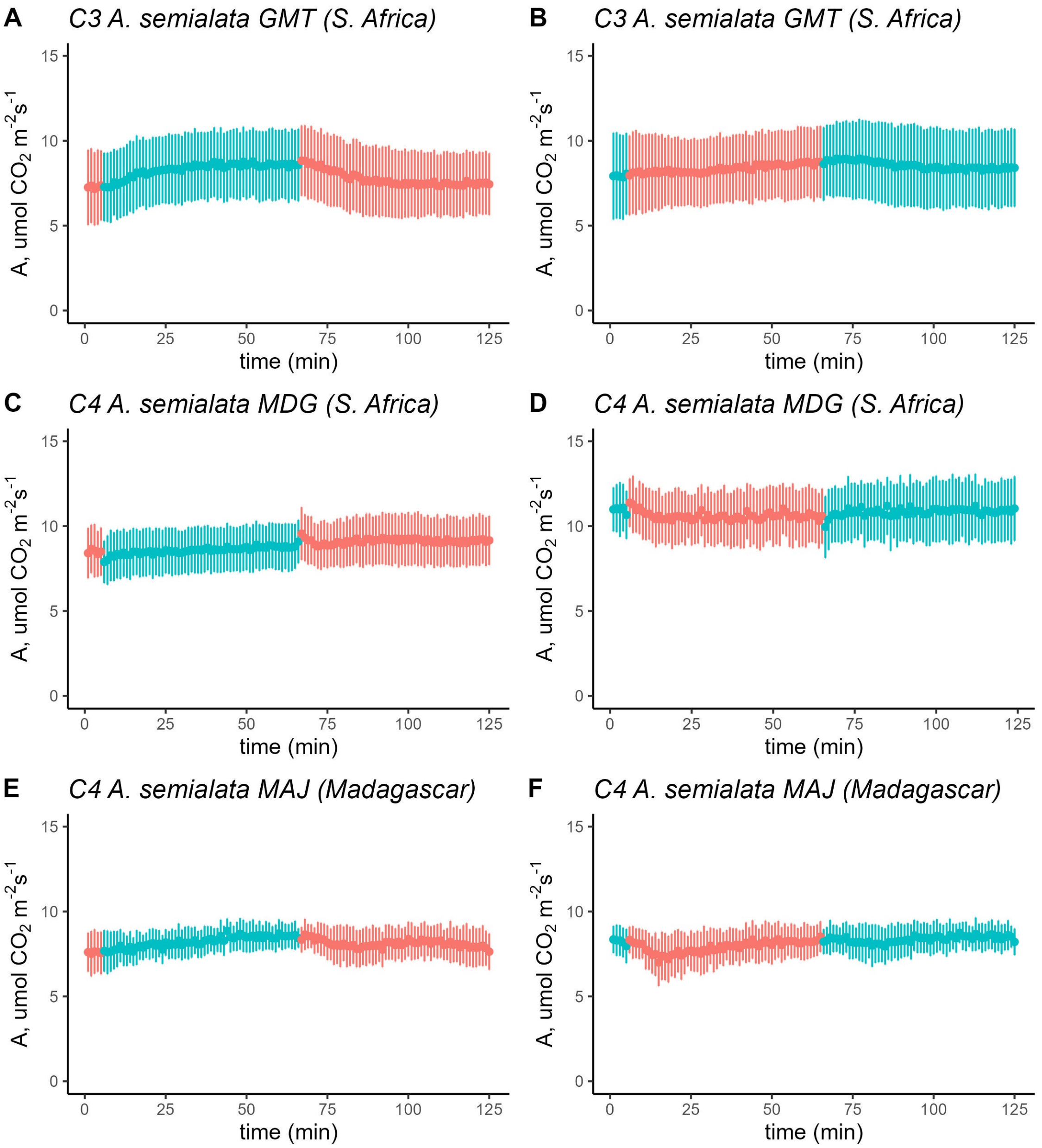

Figure S4. Time course of CO_2 assimilation (*A*) in C_3 *A. semialata* subsp. eckloniana 'GMT' (**A** and **B**), C_4 *A. semialata* subsp. semialata 'MDG' (**C** and **D**) and C_4 *A. semialata* subsp. semialata 'MAJ' (**E** and **F**) in response to a sequence of 75% red + 25% blue light and vice versa. Leaves were initially acclimated in either 100% red (**A**, **C**, and **E**) or 75% red+25% blue light (**B**, **D**, and **F**) until steady state was achieved. Subsequently, the light environment was switched depending on the initial light condition while maintaining a photosynthetic photon flux density of 500 µmol m^{-2} s^{-1}. The leaves were acclimated to the new light condition for 60 min before returning them back to the original condition for another 60 mins before terminating the experiment. Light conditions were reversed at t_6 and t_{65}. Reference CO_2 was maintained at 410 µmol mol^{-1}, block temperature was kept at 25°C and average VPD was 1.2 kPa. Data points represent mean ± se (n=3-4).

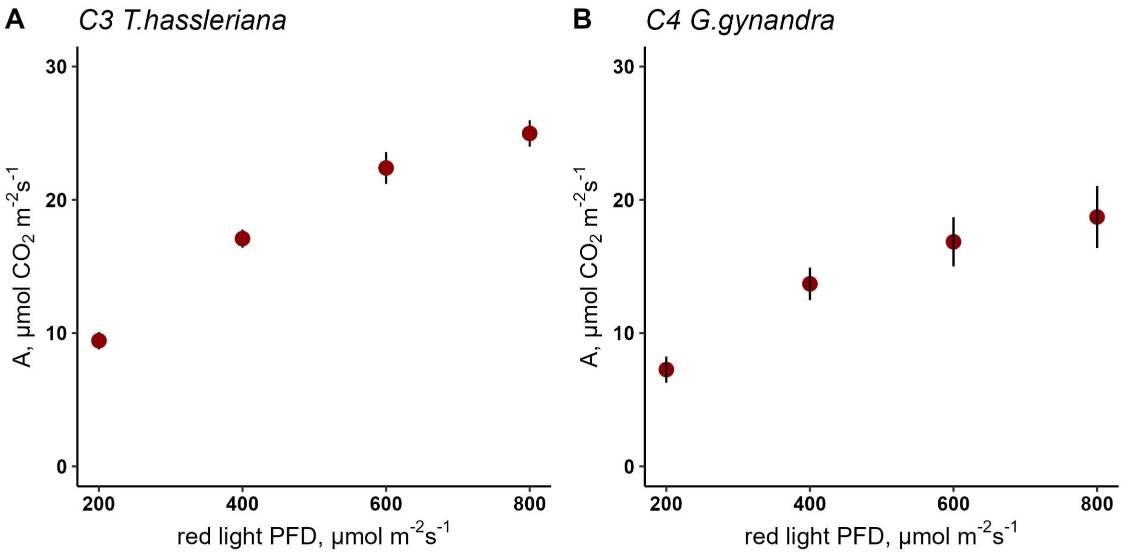

Figure S5. Response of CO_2 assimilation (*A*) to red light photosynthetic photon flux density (PPFD) in C_3 *T. hassleriana* (**A**, n=4) and C_4 *G. gynandra* (**B**, n=4). A linear mixed- effects model analysis was carried out to test if photosynthetic type influences the response to red light in congeneric species belonging to Cleomaceae. Data points represent the mean ± se.

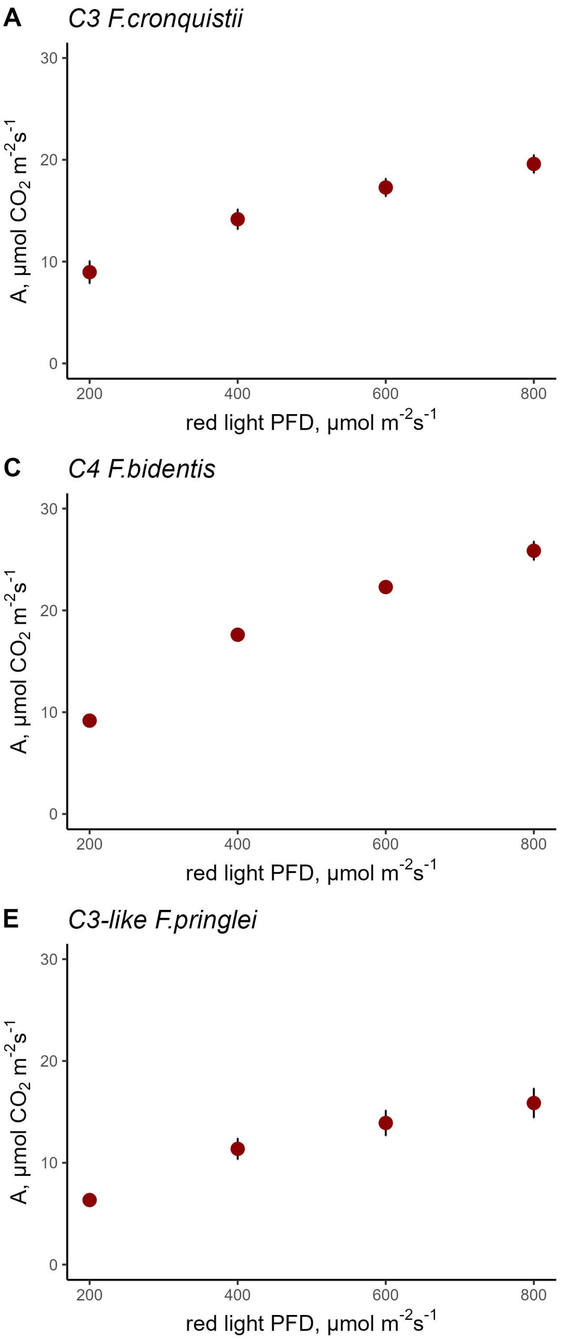

Figure S6. Response of CO_2 assimilation (*A*) to red light photosynthetic photon flux density (PPFD) in C_3 *F. cronquistii* (**A**, n=4) and C_3 *F. robusta* (**B**, n=5), C_4 *F.bidentis* (**C**, n=8), C_4 *F. trinervia* (**D**, n=5), and C_3-like *F. pringlei* (**E**, n=5). A linear mixed- effects model analysis was carried out to test if photosynthetic type influences the response to red light in congeneric species belonging to *Flaveria*. Data points represent the mean ± se.

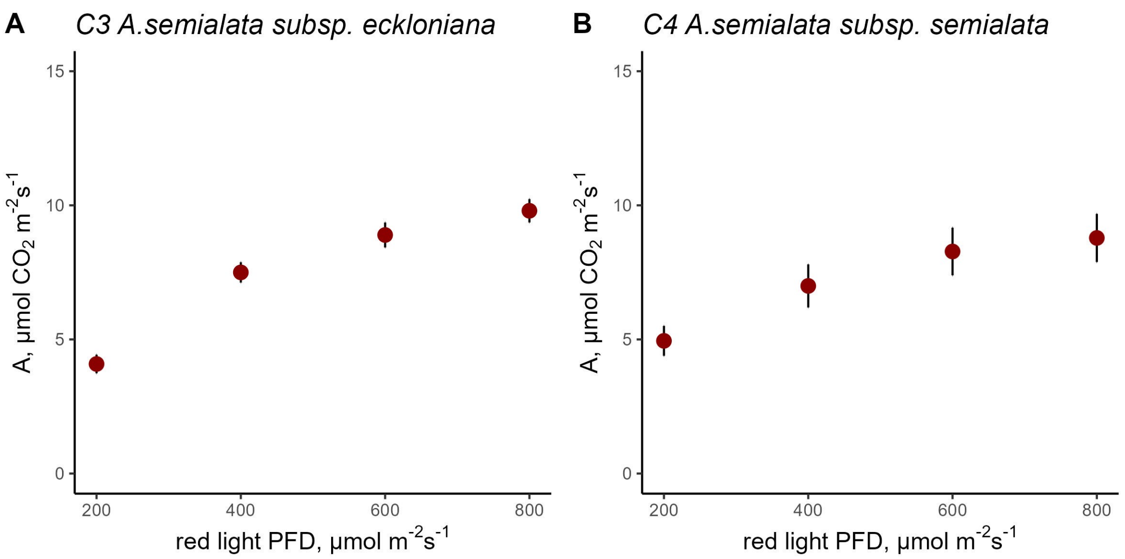

Figure S7. Response of CO_2 assimilation (*A*) to red light photosynthetic photon flux density (PPFD) in C_3 *A. semialata* subsp. eckloniana 'GMT' (**A**, n=4) and C_4 *A. semialata* subsp.semialata 'MDG' (**B**, n=5). Linear mixed-effects model analysis was carried out to test if photosynthetic type influences the response to red light in C_3 and C_4 subspecies belonging to *Alloteropsis*. Each data point represents the mean ± s.e.

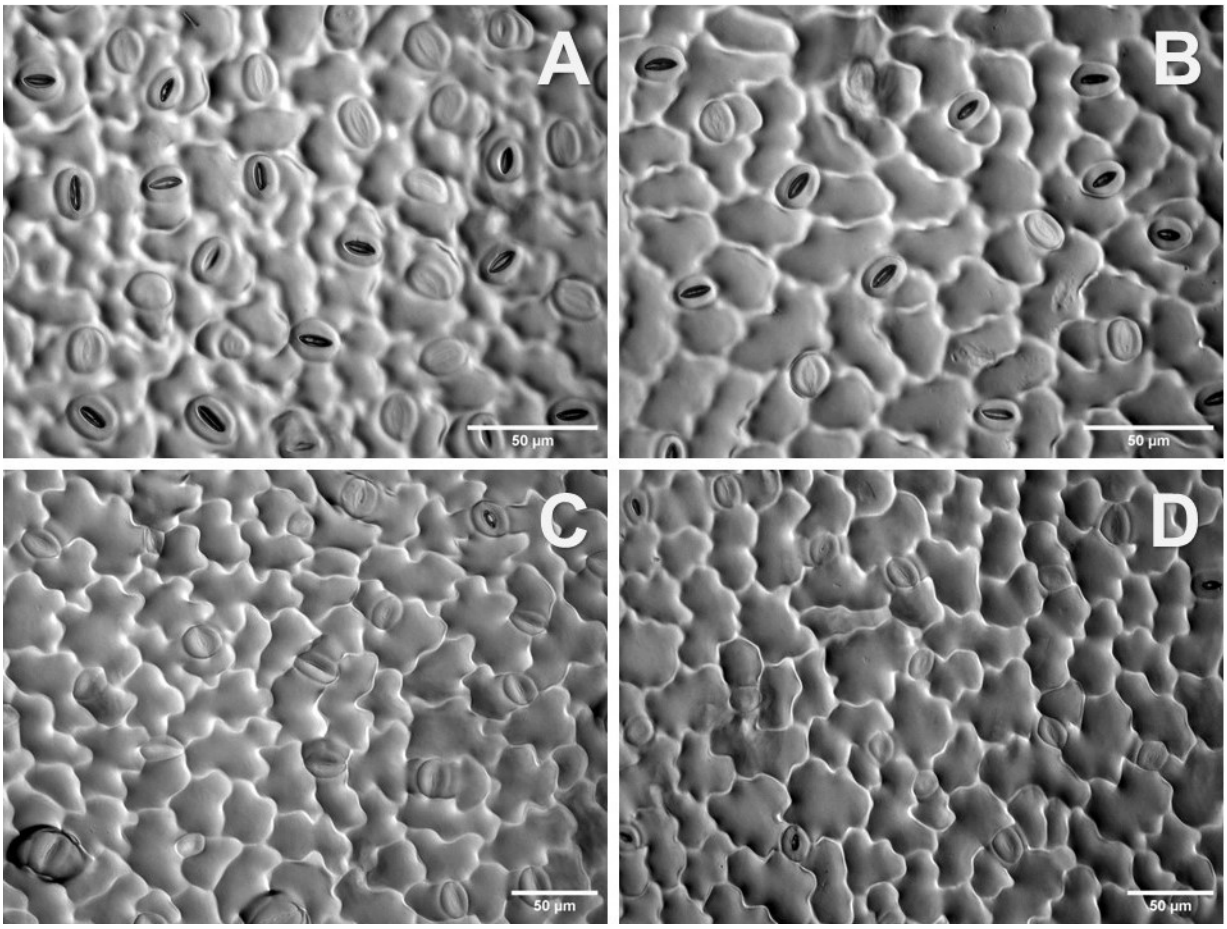

Figure S8. Nail varnish impressions from leaf surfaces of Cleomaceae species used in the study. C_3 *T. hassleriana*, (**A**) abaxial and (**B**) adaxial; C_4 *G. gynandra*, (**C**) abaxial and (**D**) adaxial. Photomicrographs were taken using Olympus microscope (BX41), at x400. Scale bar = 50 μm

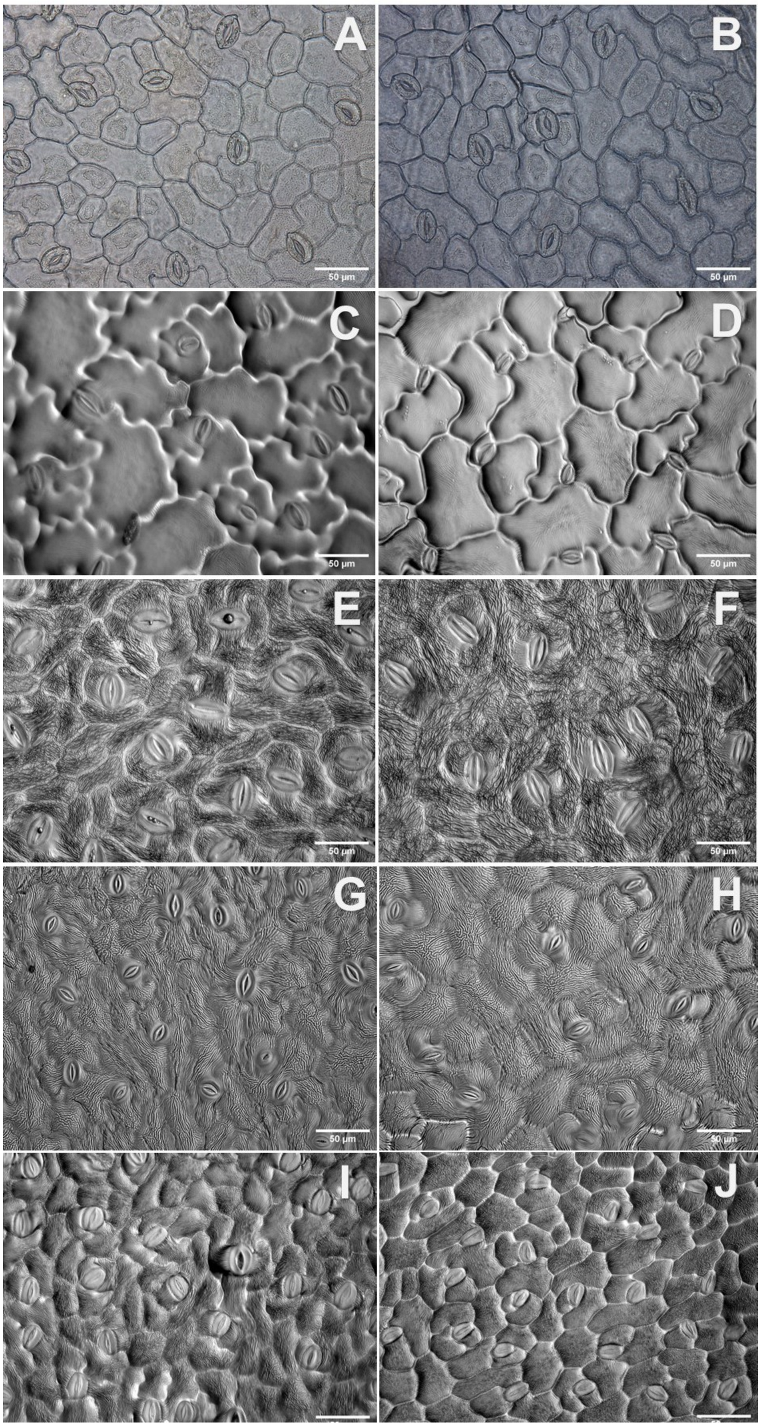

Figure S9. Nail varnish impressions from leaf surfaces of *Flaveria* species used in the study. C_3 *F. cronquistii* (**A**) abaxial and (**B**) adaxial; C_4 *F. trinervia* (**C**) abaxial and (**D**) adaxial; C_3-like *F. pringlei* (**E**) abaxial and (**F**) adaxial; C_3 *F. robusta* (**G**) abaxial and (**H**) adaxial; and C_4 *F. bidentis* (**I**) abaxial and (**J**) adaxial. Photomicrographs were taken using Olympus microscope (BX41), at x400. Scale bar = 50 μm

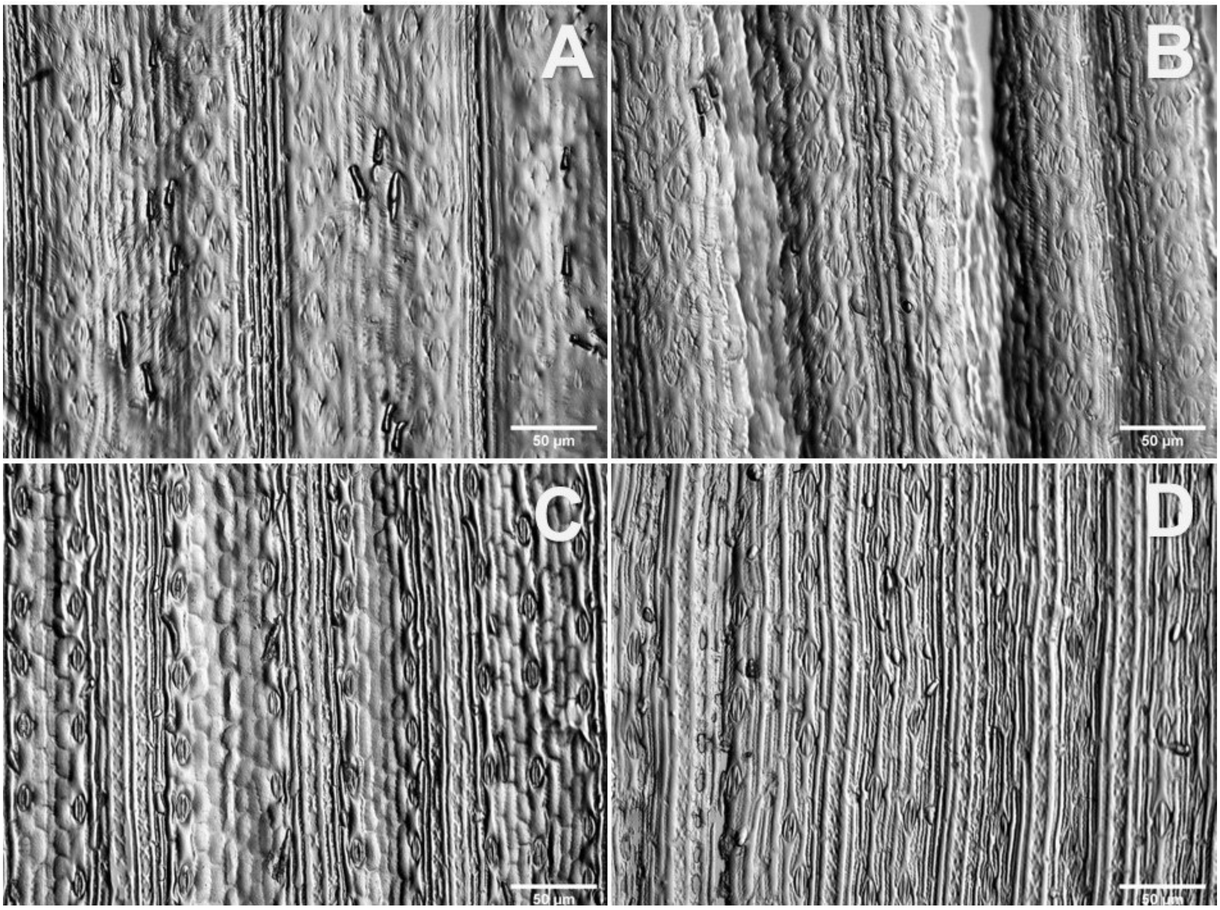

Figure S10. Nail varnish impressions from leaf surfaces of *Alloteropsis* species used in the study. C_3 *A. semialata* 'GMT', (**A**) abaxial and (**B**) adaxial; C_4 *A. semialata* 'MDG', (**C**) abaxial and (**D**) adaxial. Photomicrographs were taken using Olympus microscope (BX41), at x400. Scale bar = 50 μm
